# A virtual alternative to molecular model sets: a beginners’ guide to constructing and visualizing molecules in open-source molecular graphics software

**DOI:** 10.1186/s13104-021-05461-7

**Published:** 2021-02-17

**Authors:** Siripreeya Phankingthongkum, Taweetham Limpanuparb

**Affiliations:** Science Division, Mahidol University International College, Mahidol University, Salaya, Phutthamonthon, Nakhon Pathom, 73170 Thailand

**Keywords:** Chemical education, Molecular model, General chemistry, Molecular graphics software

## Abstract

**Objective:**

The application of molecular graphics software as a simple and free alternative to molecular model sets for introductory-level chemistry learners is presented.

**Results:**

Based on either Avogadro or IQmol, we proposed four sets of tasks for students, building basic molecular geometries, visualizing orbitals and densities, predicting polarity of molecules and matching 3D structures with bond-line structures. These topics are typically covered in general chemistry for first-year undergraduate students. Detailed step-by-step procedures are provided for all tasks for both programs so that instructors and students can adopt one of the two programs in their teaching and learning as an alternative to molecular model sets.

## Introduction

The use of molecular model has been recommended to be part of the curriculum to enhance spatial thinking in students of chemistry [1]. Physical molecular model sets are usually employed in an introductory-level chemistry laboratory [2]. Other similar teaching tools [3–45] vary greatly in terms of cost, ease of use, required amount of time and suitability for relevant topics in chemistry. We have considered and tried some of them in our class and arrived at some observations. Table 1 shows a brief review of the literature and our observations on some physical molecular models.

**Table 1:** A brief review of molecular model teaching tools and our observations on some physical molecular models

Molecular Model Teaching Tools	Our observations
**Physical molecular models** o Ping-pong balls [3], rubber balloons [4], bottle caps [5, 6], whiteboard markers [7] or other materials [8–14] (Repurposing available materials) o Plasticine [15], clay or dough [16], (Hand-building from malleable materials) o Laser/wire cut [17], 3D printing [18–21], 3D Laser engraving [22], magnet-embedded silicone [23] (Custom-made models by advanced techniques) **Computer software or web apps** o Specialized online services such as molecular clickers based on 3Dmol.js [24], Android application for chemical OCR and 3D visualization [25], unit cells modelling [26] and molecular shape modelling [27] o Platonic solids in POV-Ray language [28] or orbital displays in Mathematica [29] o Molecular graphics software o Proprietary commercial software e.g. GaussView [30], Chem3D [31] o Open-source software e.g. Avogadro [32, 33], Chimera [34], Gabedit [35], IQmol [36], PyMOL [37] and QuteMol [33] o Online service for molecular graphics such as WebMO [38] and CheMagic [39] **3D/augmented reality** (Advanced visualization techniques usually require both specific hardware and software for operation.) o 3D video glasses [40, 41] o App on mobile devices [42–44] o Holographic projection [45]	● Commercial molecular model sets can be a costly one-time investment. As many pieces are included in a set to represent various atom/bond types, molecular model users usually spend a considerable amount of time for inventory inspection. The time may be reduced if an analytical balance is used for this counting process. In many cases, students also spend a significant amount of time or find it difficult to disassemble models back to the original pieces. This disassembling is usually needed before building the next molecules as a standard molecular model set of 165 pieces contains only 14 carbon atoms. ● Other physical models (such as table tennis balls and plasticine) may have pros and cons. For example, plasticine is malleable and can be used to form ball-and-stick as well as space-filling models. We asked students to weigh plasticine so that they hand-build models that are proportional to actual molecules. Students clearly observed the relative size of hydrogen compared to halogen in hydrogen halides and correctly predicted the acidity trend. However, the use of plasticine can be cumbersome, dirty and distracting for some students.

Molecular graphics software has been available in the market for many decades. Until recently, these programs were intended for sophisticated users of quantum and molecular mechanics modelling and were licensed at high price. With open-source licensing, the cost and availability of the software is no longer an issue.

Most molecular graphics programs allow users to construct, edit and visualize molecules in 3D, and hence, are viable alternatives to conventional teaching materials. The issue here is therefore how to bring these dynamic and interactive visualization programs as scaffolding tools for student learning [46]. A number of papers have described the use of these software for upper-level undergraduate teaching [24, 25, 31, 33, 34, 47, 48]. However, educational exercises of introductory-level chemistry are limited as these programs are regarded as specialized and used mainly for research.

This paper describes our experience in using two programs, Avogadro and IQmol, for a lesson in an introductory-level chemistry laboratory class. The lesson was designed to maximize alignment to the content taught in general chemistry. Advanced features traditionally associated with using these programs are intentionally left out. To the best of our knowledge, our work is the first paper that explicitly describes educational uses of molecular graphics software for this audience level.

The laboratory course is the first laboratory in chemistry for science students. The class can be taken independently of the lecture class. The aims of this four-hour session with the software are

1. to use the virtual tool in the same way that physical molecular models are used (Tasks 1 and 4 below)
2. to use some additional visualization features of the software related to those discussed in general chemistry course without running a quantum chemical calculation (Tasks 2 and 3 below).

Instructions are designed so that students can easily follow even without prior knowledge in chemistry and students can also appreciate producing textbook-quality pictures. Due to the clear separation of laboratory and lecture in the curriculum, we do not intend to teach the underlying concepts but only provide tools and practices to support their learning.

### Description of software and features

Avogadro [32] and IQmol [36] are open-source and cross-platform for both Mac and Windows, launched in 2016 and 2015 respectively. Both programs are light-weight and easy to install. They offer a range of features to run and analyze calculations. Each program requires minimal disk space (< 50 MB) and, for most users, downloading and installing can take less than 5 min. The programs are also available for Linux but the installation may not be trivial.

As mentioned earlier, in this paper, we do not intend to introduce beginners to run any calculations [47–49] or work with any macromolecules. We use only the following abilities of the programs.

o Read different types of files (xyz or z-matrix geometry, check point file, frequency calculation output).
o Build and edit molecules by graphic user interface and optimize structures by built-in molecular mechanics force fields.
o Visualize molecules in various representations (ball-and-stick or space-filling) as well as displaying bond length, bond angle and torsional angle.
o Some of the advanced features e.g. displaying animations, dipole-moment, spectra and surfaces (orbitals and densities).

## Main text

### Student tasks and implementation

The computer-delivered molecular model activity has been adopted in a first-year chemistry lab at Mahidol University since 2019. Student worksheet in Additional file 1 was developed and continually revised. Students were asked to complete a two-page worksheet that comprises of four sets of tasks. The total time to complete all tasks is approximately three to four hours. Our activity is delivered in a computer lab during the first week of the course as students are yet to find their own lab coat and goggles for a chemical laboratory. As a result, the safety induction/demonstration session [50] is scheduled in the second week and the real experiment commences in the third week. This provides more time for students and staff to complete necessary arrangements and preparation associated with the laboratory class.

We encourage students to use one of the two programs installed in advance at our computer lab. However, some students want to use their own laptops. It is perfectly legal to download/share and install these open-source programs on student machines.

Students perform differently on our tasks depending on their computer literacy. Assigning them randomly into groups of two in the same way as other laboratory assignments partially helps with the difference. It is best to demonstrate one example from each task for four tasks consecutively. We make sure that all groups are able to perform the example tasks correctly before letting them work independently. Students are also welcomed to show different approaches to accomplish the tasks. (Screenshots of steps to perform these tasks are in the Mendeley Data Repository.)

Optional exercises highlighted in yellow in Additional file 2 are also discussed below for these tasks. While the main tasks require little or no prior knowledge from the lecture class, these optional exercises require some background knowledge. They are usually more challenging and time-consuming. These exercises were used in our previous version of the worksheet but no longer used in our current version due to time constraint and their difficulty. We recommend that, if desired so, only some of these exercises are included for advanced learners as appropriate for the class. The objective of incorporating optional exercises to every task was to provide learners with stronger link from the tasks to chemistry topics.

### Task 1: Building basic molecular geometries

**Fig. 1:**
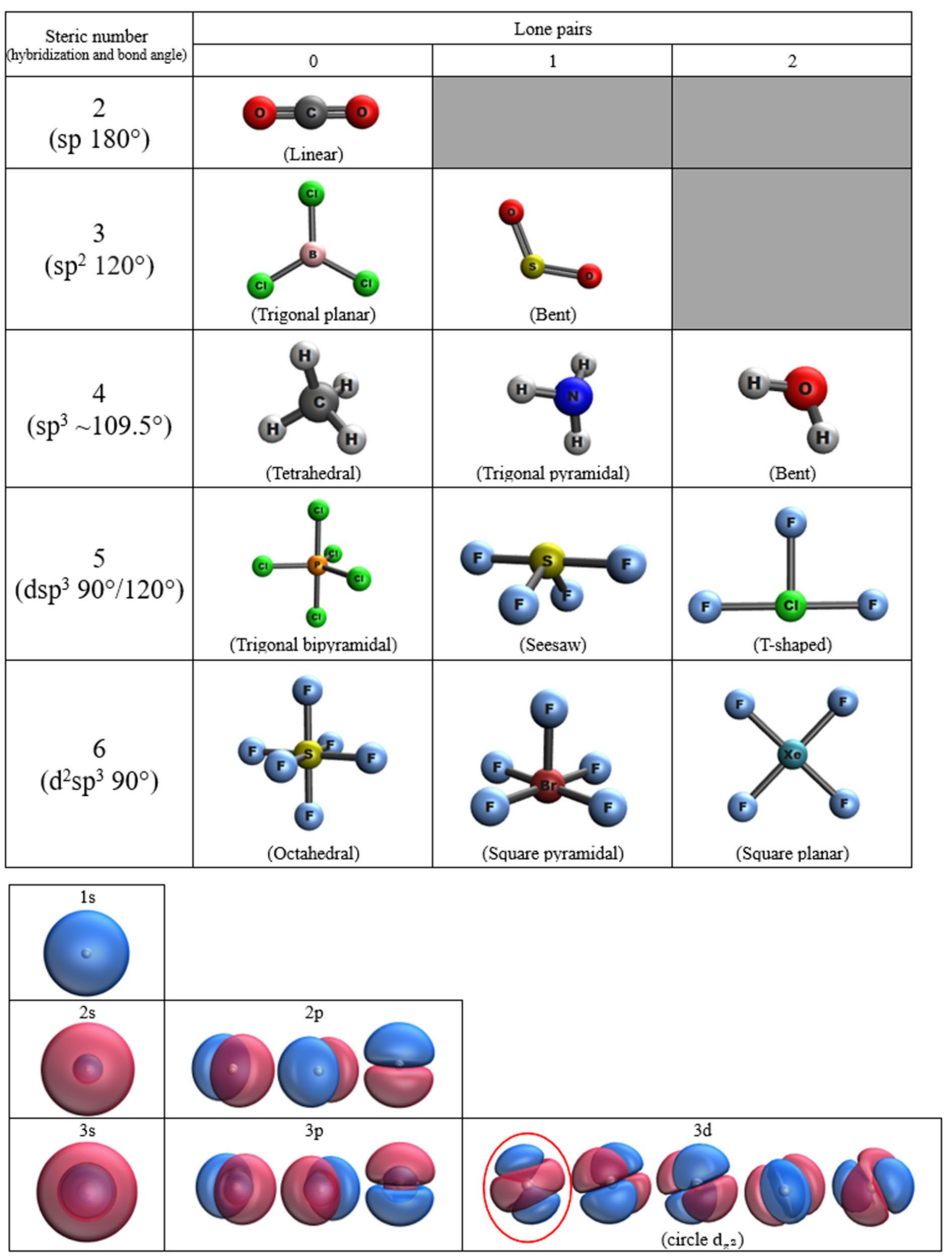
A sample work of Tasks 1 and 2 in which students build molecules from various templates and visualize hydrogen-atom orbitals in IQmol

Students are asked to produce ball-and-stick representation of 12 single-center molecules on the worksheet as shown in Fig. 1. The molecules are typical examples of structures covered in the VSEPR theory. In this task, students pick a xyz file of the template molecule with a steric number ranging from two to six and delete/replace atoms as appropriate. This building-by-template approach allows the software to operate in the same way as a physical molecular model set. All bond lengths and angles do not change as students build a new molecule by deletion or substitution of atoms from the template.

The optional exercise comprises of various problems. Find the origin of each bond angle in the table including the angle of ~ 109.5° for a tetrahedral structure. Find structure of an additional molecule for VSEPR theory. Render a molecule in space filling model. Apply force field to molecules and use Bent’s rule to explain deviation from the expected bond angle of ~ 109.5° in ammonia and water and slight difference in C-H bond length in methane and halomethanes [51].

### Task 2: Visualizing orbitals and densities

Two checkpoint files are prepared in advance for students to render surfaces in the software. Molecular orbitals (MOs) such as highest occupied molecular orbital (HOMO) and lowest unoccupied molecular orbital (LUMO) are explored. Students use the first file to visualize atomic orbitals (1s, 2s, 3s, 2p, 3p and 3d) of a hydrogen atom. The second file of formaldehyde is used to construct HOMO, LUMO and the total electron density map of a molecule. Students appreciate the shapes and relative sizes of atoms and molecules from the density map.

The optional exercise is to observe the effect of different isovalues on the results and explore other rendering features such as mesh rendering and clip.

### Task 3: Predicting polarity of molecules

Students build a molecule, establish a colored-electrostatic potential map, show partial charges on atoms and display a dipole-moment arrow on each molecule. This helps students understand the concepts of electronegativity, resonance structure, bond dipole moment and polarity of molecules.

The optional exercise is to explore vibrational spectroscopy from a given quantum chemical calculation output file. Students can view the vibrational mode of a molecule at different frequencies in an animation as well as observe its Infrared/Raman spectrum. Not only that the task allows students to enjoy the interactive feature of the software, it links the concept of dipole-moment to the selection rule in vibrational spectroscopy.

### Task 4: Matching 3D structures with bond-line structures

A collection of geometry files is given to students to open with the program. Students match each 3D visualization on the program to 2D bond-line structure e.g. Fischer projection, Newman projection, chair conformation. Students can write down file name(s) below these structures in the tables in the worksheet.

The optional exercise comprises of identifying all enantiomers, diastereomers and rotamers along with ranking their stability; changing a chair structure of a substituted cyclohexane to the other chair structure; and building more complex molecules such as paracetamol and aspirin. Additionally, single-letter file names in the main matching task above can be replaced by systematic or common names. In the process, students will be familiarized with the names of these chemical structures.

## Results and discussion

We have designed a new laboratory exercise for beginner students of chemistry to learn molecular modelling. The use of computer programs for molecular model lesson helps overcome traditional difficulties and offers more flexibility in teaching and learning activities. A significant portion of our tasks requires students to document their work by putting images in the worksheet. These images are more or less the same as figures in most general chemistry textbooks and help students prepare for or reinforce chemical concepts in lecture classes. We have limited evidence that students later use the skills acquired here to produce high-quality image for later tasks in the class [52, 53].

While these tasks are intended to be delivered face-to-face, during the period of COVID-19 outbreak, the tasks were completed remotely in May 2020 and January 2021 with minimal modifications. The instructor (TL) and a teaching assistant (SP) provided instruction via live streaming to students. Students still worked in pairs and submitted one worksheet per pair during the class as we normally do for an on-campus class. A few of our students reported a technical difficulty with one of the two programs at home. We then suggested that they try the other program to resolve the issue.

Pre- and post-tests were used in an on-campus class during September 2020 to assess student learning during the laboratory exercise. Average student score in a class of 31 students were increased from 63% to 74% after working on the activities. Plus/minus/delta questions were part of the post-test. Detailed comments can be found in Additional file 3. In brief, students use the following words to describe their positive experience, like (*n* = 8), see (*n* = 9), visualize/visually (*n* = 5), interesting (*n* = 2) and understand (*n* = 2). The area for improvement includes time management for the class and pace of instruction.

In conclusion, repurposing the two freely available molecular graphics programs, Avogadro and IQmol, from specialized uses to educational uses for beginners as described in this paper are viable alternatives to conventional molecular model sets. These simple exercises can be used in other class settings, other available programs or online services [38, 39] with minimal effort. Moreover, these activities can be easily delivered online to comply with physical distancing requirements during the pandemic.

## Limitations

- Some concepts such as bond order and resonance may not be well-represented in the programs. Other concepts such as formal charge on atom, total charge of the structure, and non-bonding electron may not be available for visualization.
- The quantitative evidence of learning in this paper is preliminary (two-tailed *p*-value = 0.055 for an on-campus class in September 2020) and may be influenced by taking the parallel tests twice (in pre- and post-tests).
- To avoid plagiarism, students may be asked to produce videos by a built-in screen recorder (XBox on Windows or Quicktime on Mac) to show the process of their work.
- Installation of the programs may be limited by a security policy. “The developer cannot be verified” is a known issue which can be bypassed.

## Supplementary Information

**Additional file 1.** Worksheet and files for students.

**Additional file 2.** Grading criteria and complete solutions inclusive of optional exercises.

**Additional file 3.** Survey questions and detailed results.

## Data Availability

The datasets supporting the conclusions of this article are available in the Mendeley Data repository, https://doi.org/10.17632/hfynpvtrz3: Manual containing screenshots of step-by-step instruction on how to complete the assigned tasks and example videos of the process.

## Abbreviations

OCR: Optical Character Recognition
POV-Ray: The Persistence of Vision Ray Tracer
VSEPR: Valence shell electron pair repulsion theory
