# Supplementary material for: A virtual alternative to molecular model sets: a beginners’ guide to constructing and visualizing molecules in open-source molecular graphics software
Source: BMC Res Notes. 2021 Feb 17;14:66. doi: 10.1186/s13104-021-05461-7 (PMC7887714; doi:10.1186/s13104-021-05461-7)
Supplement: Supplementary file 1 — Additional file 1. Worksheet and files for students. [file 13104_2021_5461_MOESM1_ESM.zip › Manual/Manual.pdf]

Unlisted video instruction on youtube.com

https://www.youtube.com/playlist?list=PLNbV5znV1KFuRzOXltx7-WsRjv-j4Pd0W

A Virtual Alternative to Molecular Model Sets:

A Beginners' Guide to Constructing and Visualizing Molecules in Open-source Molecular Graphics Software

DOI for the main paper: 10.1186/s13104-021-05461-7 DOI for this manual: 10.17632/hfynpvtrz3

Table of Content

AVOGADRO (1.2.0) 3

Task 1: Building basic molecular geometries **4**

Task 2: Visualizing orbitals and densities **5**

Task 3: Predicting polarity of molecules **7**

Task 4: Matching 3D structures with bond-line structures **9**

IQMOL (2.14) 10

Task 1: Building basic molecular geometries **11**

Task 2: Visualizing orbitals and densities **13**

Task 3: Predicting polarity of molecules **15**

Task 4: Matching 3D structures with bond-line structures **17**

DOI for the main paper: 10.1186/s13104-021-05461-7 DOI for this manual: 10.17632/hfynpvtrz3

Avogadro (1.2.0)

- Avogadro: an open-source molecular builder and visualization tool.
  Version 1.2.0. http://avogadro.cc/
- Marcus D Hanwell, Donald E Curtis, David C Lonie, Tim Vandermeersch, Eva Zurek and Geoffrey R Hutchison; "Avogadro: An advanced semantic chemical editor, visualization, and analysis platform" *Journal of Cheminformatics* **2012**, 4:17.
- Avogadro is available under the GNU General Public License (GPL).

Task 1: Building basic molecular geometries

Constructing molecules

Five molecular structures in the first column of the table have been given as templates to obtain the rest of the assigned molecule. Each of the given molecules is the first molecule of the row, which serves as a template for the remaining molecule(s) in the row (e.g. BCl_3 is a template molecule for SO_2).

- Open the given template file with Avogadro
- Select **Selection Tool** icon in the tool bar → Select a bond or an atom wished to delete and press **delete** key on your keyboard
- To delete multiple parts of the molecule at the same time → Hold the **shift** key on your keyboard while selecting the parts wished to delete → Press **delete** key

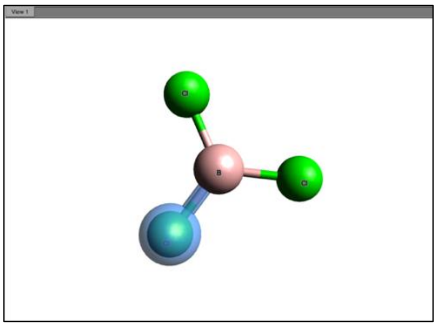

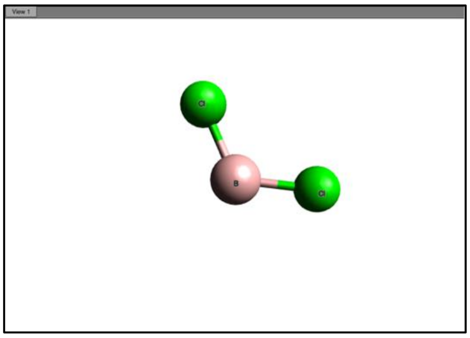

- Select **sulfur (S) element** under **Draw Settings** → Click on the boron (B) atom in the viewer window to replace it with sulfur

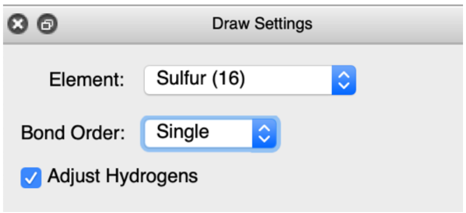

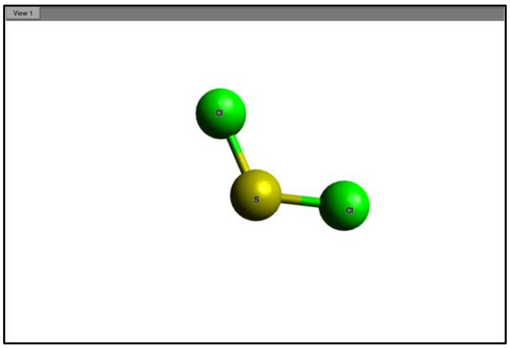

- Similar to the previous step, select **oxygen (O) element** under **Draw Settings** → Click on the two chlorine (Cl) atoms in the viewer window to replace them with O atoms

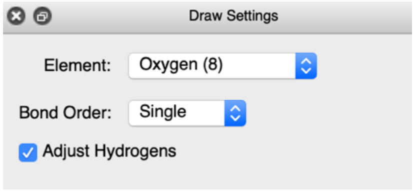

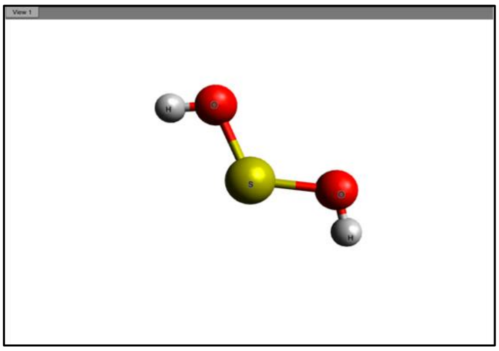

DOI for the main paper: 10.1186/s13104-021-05461-7 DOI for this manual: 10.17632/hfynpvtrz3

- Change the **Bond order** by selecting **Double** to form a double bond under **Draw Settings** → Click on the two single bonds between S and O

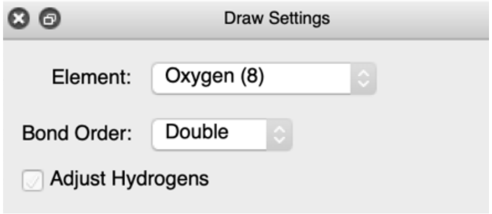

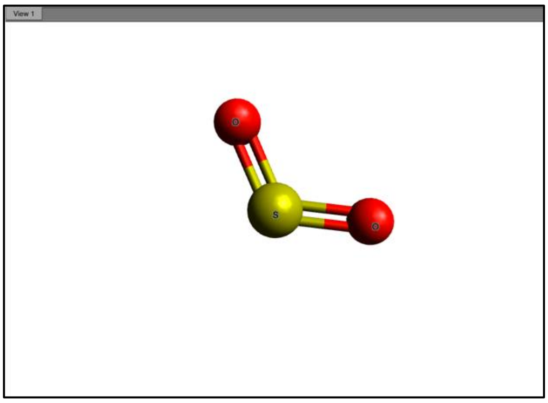

Task 2: Visualizing orbitals and densities

Mapping Electron Density

- Select **File** → Select **Open** → Open the given checkpoint file, **CH2O.fchk**

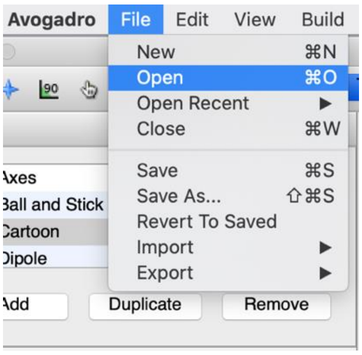

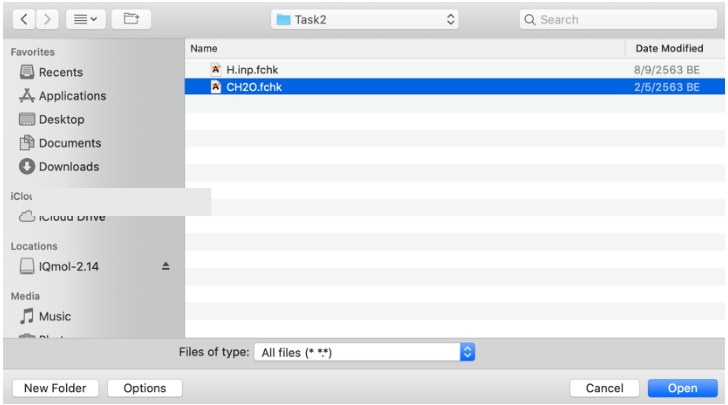

- Select **Extension** → Select **Create Surfaces** → For **Color By**, select **Electron Density** → **Calculate**

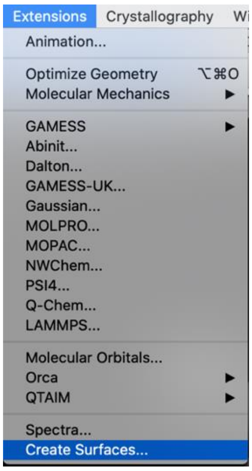

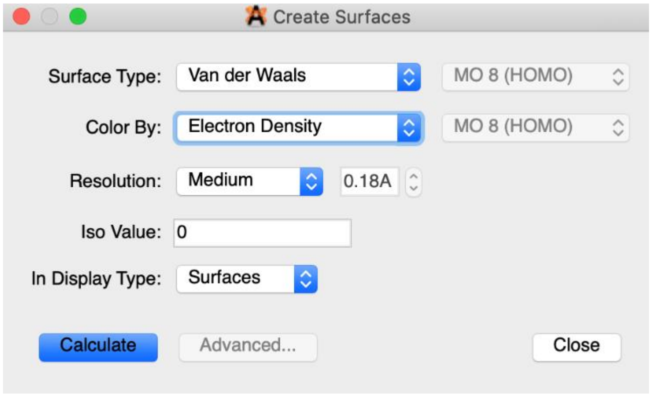

To display the surface clearly, change the background color to white:

- Select **View** → Select **Set Background Color…** → Select white color

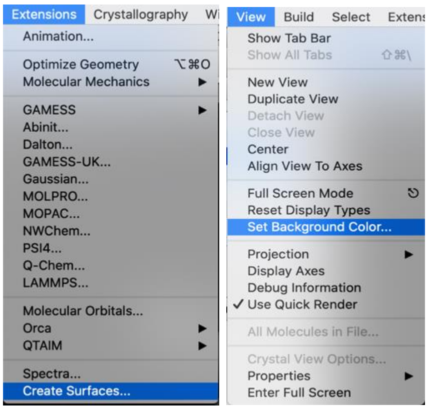

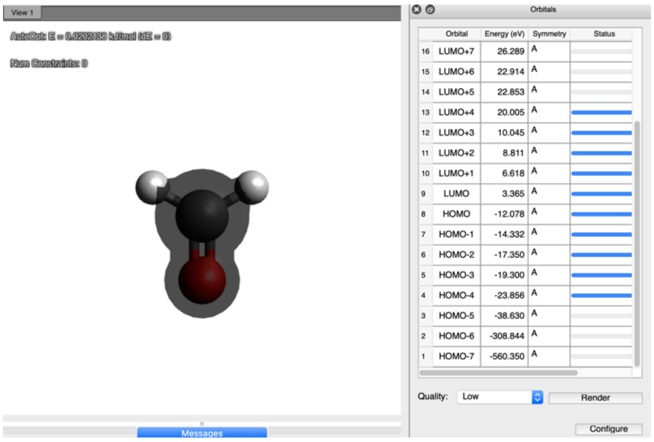

Color the surface for clearer observation and interpretation:

- In Display Types, select the wrench icon 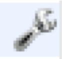 beside **Surfaces**
- Under **Surface Settings**, for **Render** → Select **Fill** → Set **Positive** as blue color and **Negative** as red color

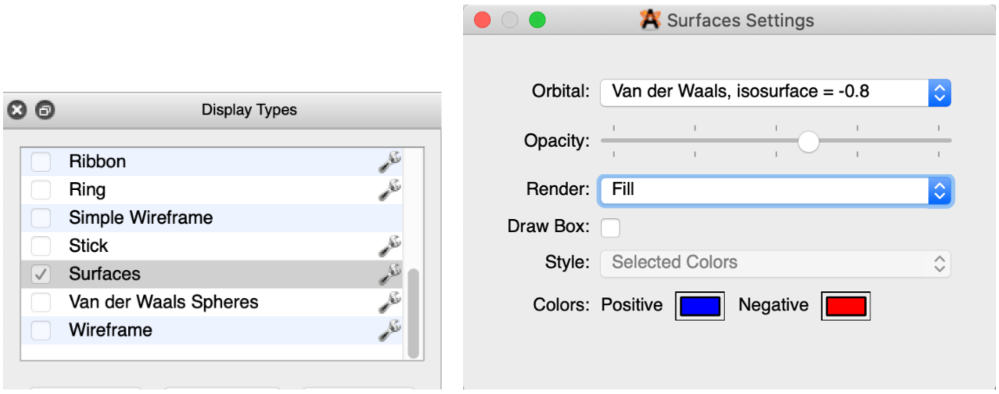

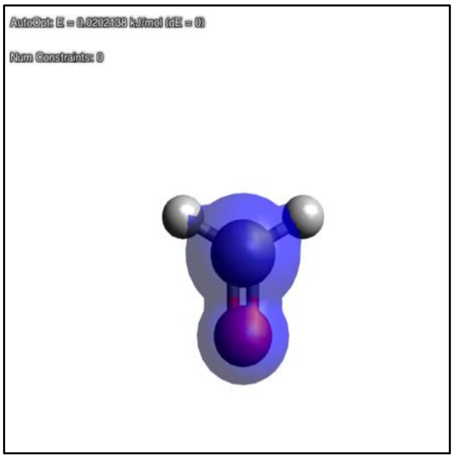

DOI for the main paper: 10.1186/s13104-021-05461-7 DOI for this manual: 10.17632/hfynpvtrz3

Generating Molecular Orbitals (HOMO and LUMO)

HOMO

- Make sure the blue status bars are completely loaded
- Under **Orbitals,** Select **HOMO**

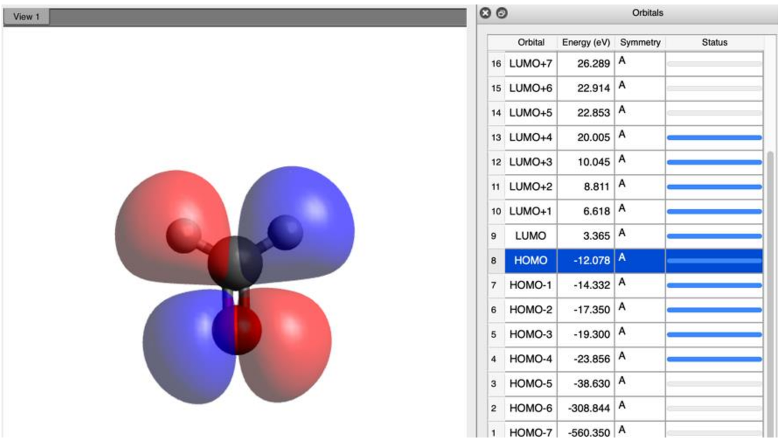

LUMO

Same steps can be followed, except for selecting **LUMO** under **Orbitals** instead of HOMO

Task 3: Predicting polarity of molecules

Part A: Generating Electrostatic Potential Map and Displaying Dipole-moment

- Build a H_2O molecule by selecting **Draw Tool** icon 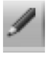 → Under **Draw Settings**, select an element and bond order you wished to build. Click on an atom and drag to form a bond → Select **Auto Optimization Tool** 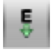 to minimize the energy of the molecule → Under **AutoOptimization Settings**, select **Start** and select **Stop** after the optimization process is completed

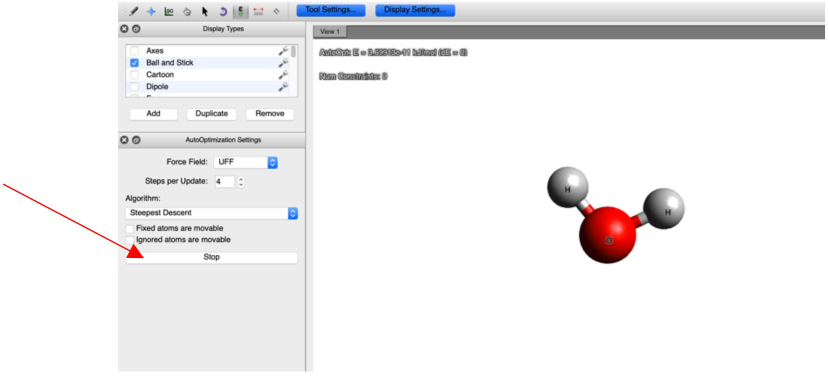

DOI for the main paper: 10.1186/s13104-021-05461-7 DOI for this manual: 10.17632/hfynpvtrz3

- Select **Extension** → Select **Create Surfaces…** → For **Color By**, select **Electrostatic Potential** → **Calculate**

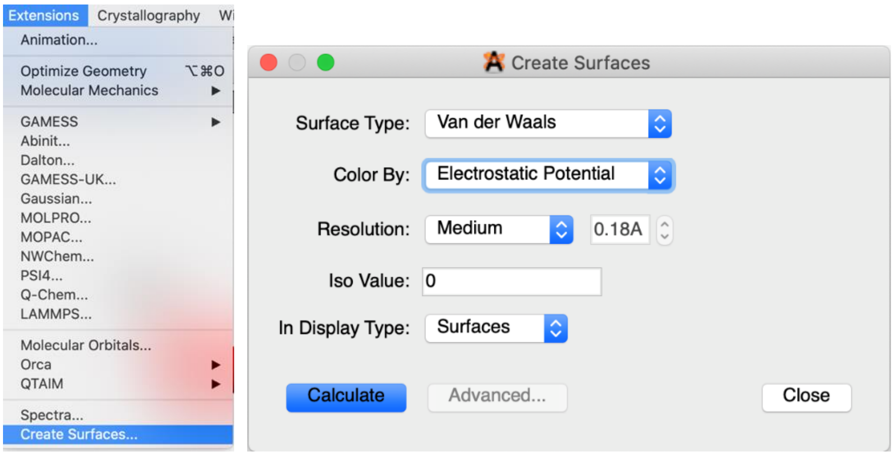

- Under Display Types, select the **Dipole** checkbox to display the dipole-moment vector

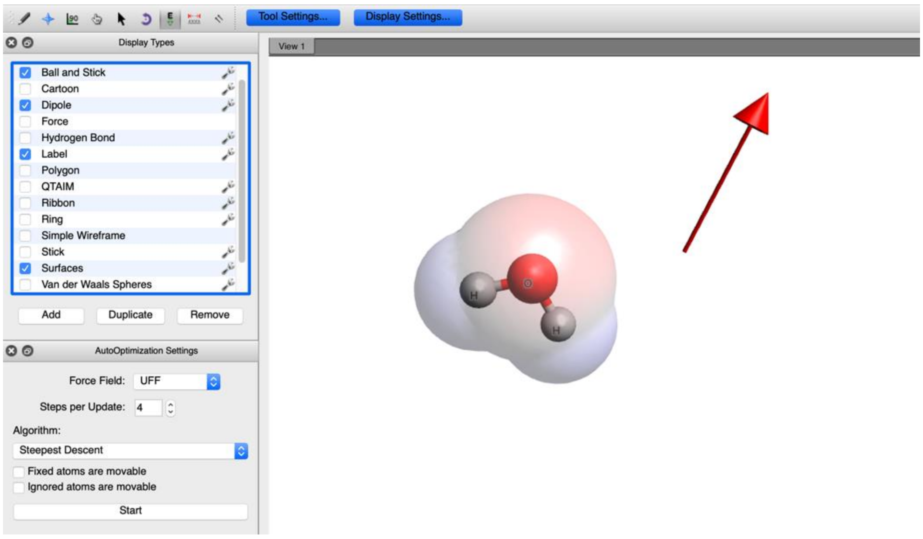

- To change the origin of the dipole vector, select the **wrench icon** 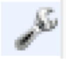 beside **Dipole**

DOI for the main paper: 10.1186/s13104-021-05461-7 DOI for this manual: 10.17632/hfynpvtrz3

Task 4: Matching 3D structures with bond-line structures

- Select **File** → Select **Open**

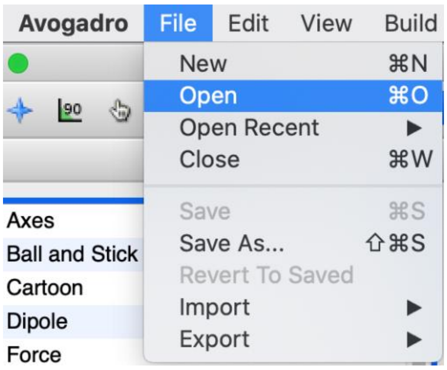

- Open each of the files in folder, **Task4** to display the 3D structure of each molecule

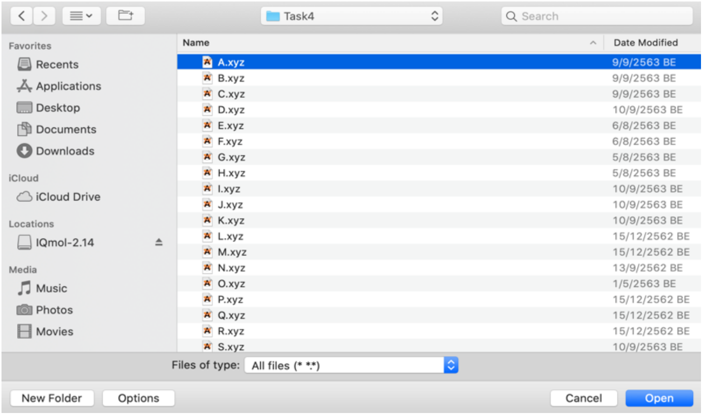

- Select **Manipulation tool** icon 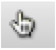 or 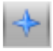 in the tool bar to adjust the perspective of the molecule

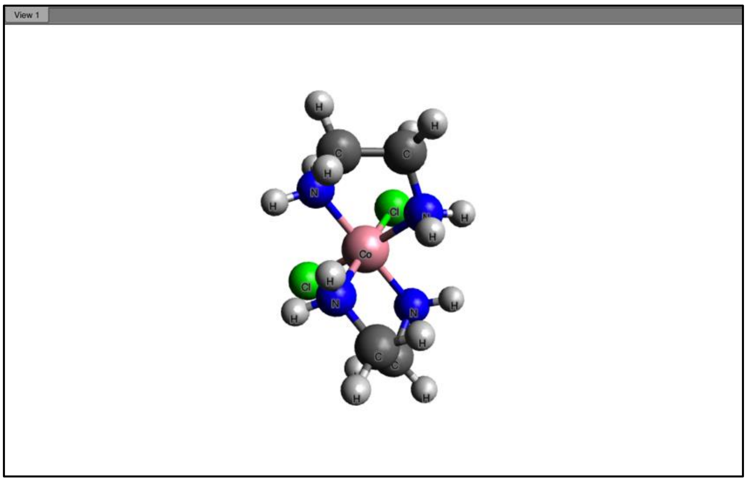

IQMOL (2.14)

- IQmol, a molecular builder and visualization package is written by Andrew Gilbert. IQmol is able to build molecules, set up and submit input for Q-Chem calculations, and analyse the output. Analyses include display of molecular surfaces (densities, molecular orbitals) and animations of frequencies and reaction pathways.
- For an up-to-date list of features and pre-compiled binaries, please visit the website: http://iqmol.org
- IQmol is available under the GNU General Public License (GPL).

DOI for the main paper: 10.1186/s13104-021-05461-7 DOI for this manual: 10.17632/hfynpvtrz3

Task 1: Building basic molecular geometries

Constructing molecules

Five molecular structures in the first column of the table have been given as templates to obtain the rest of the assigned molecules. Each of the given molecules is the first molecule of the row, which serves as a template for the remaining molecule(s) in the row (e.g. BCl_3 is a template molecule for SO_2).

- Open the given template file with IQmol
- To display the element name, go to **Display** → **Atom Labels** → Select **Element**

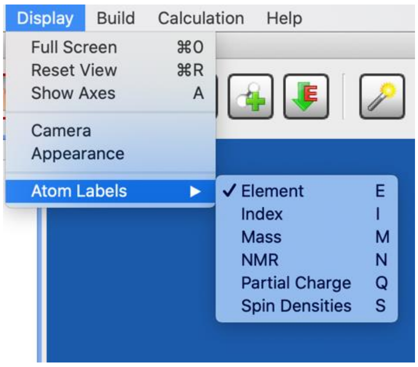

- Select **Selection Mode** icon 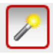 in the tool bar → Select the bond(s) and atom(s) wished to delete → Select the **Delete Selection** icon 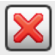 in the tool bar

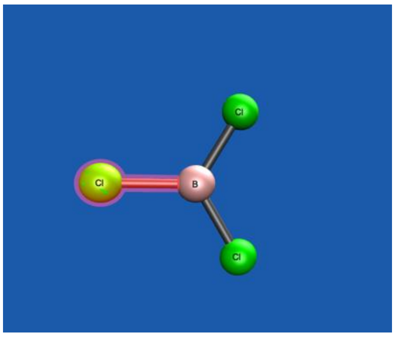

- Select **Build Element** icon in the tool bar → Select Sulfur (S) atom 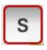 → Click on the boron (B) atom in the **Viewer window** to replace it with sulfur

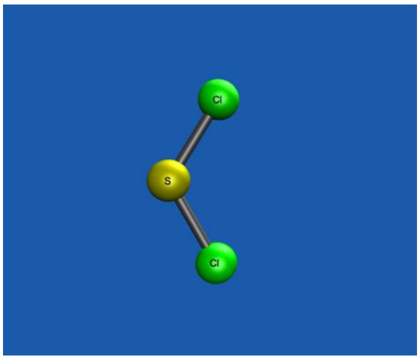

DOI for the main paper: 10.1186/s13104-021-05461-7 DOI for this manual: 10.17632/hfynpvtrz3

- Similar to the previous step, select **oxygen (O) atom** 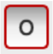 by selecting **build element** icon → Click on the two chlorine (Cl) atoms in the viewer window to replace them with O atoms

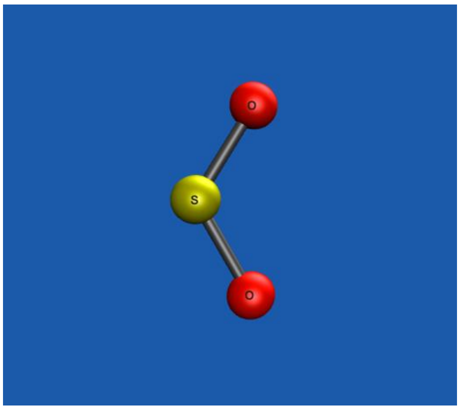

- **Click** on the sulfur (S) atom once again and **drag** to the oxygen (O) atom. Repeat this step for the other S-O bond to increase the bond order

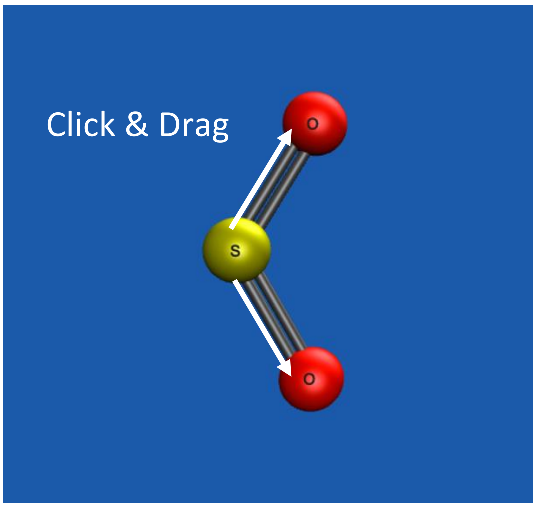

DOI for the main paper: 10.1186/s13104-021-05461-7 DOI for this manual: 10.17632/hfynpvtrz3

Task 2: Visualizing orbitals and densities

Generating Atomic Orbitals of Hydrogen atom

- Select **File** → Select **Open** → Open the given checkpoint file, **H.inp.fchk**, under Task2

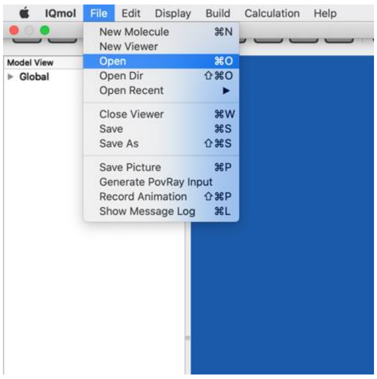

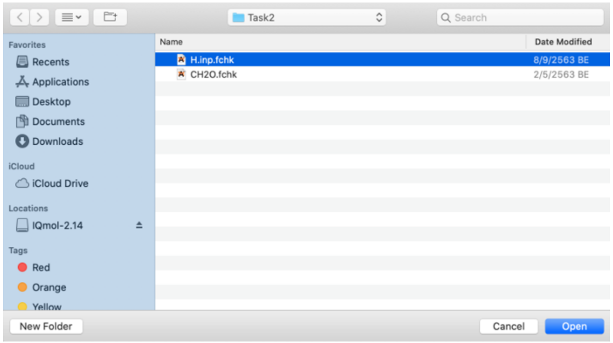

- In the **Model** View → Under **Surfaces**, double click **Canonical Orbitals** → For **Orbital(s)**, select **Alpha (HOMO)** to **Alpha 30** to view all atomic orbitals of H → **Calculate**

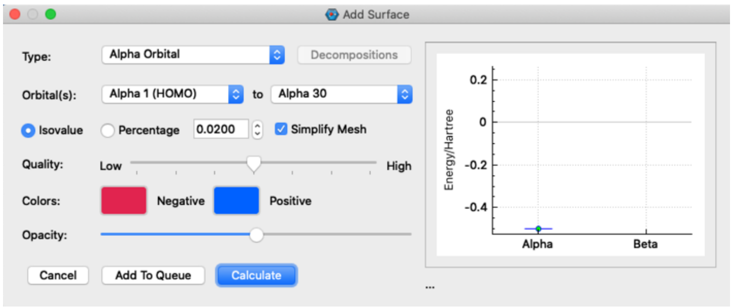

- Under **Canonical Orbitals** → Select the checkbox next to the orbital's name to view and unselect the checkbox to hide it

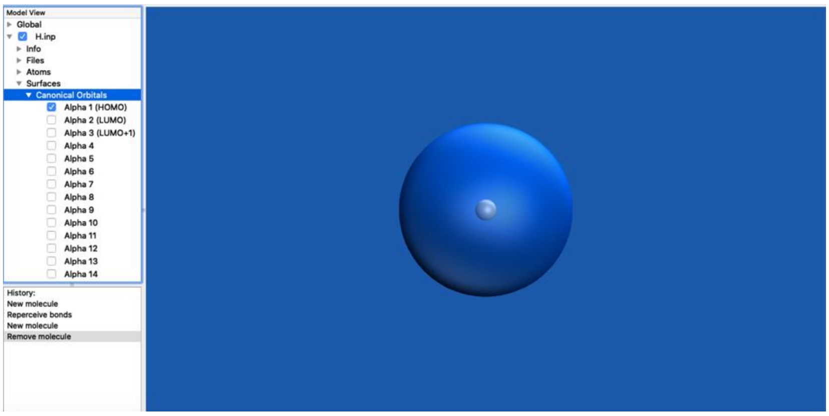

DOI for the main paper: 10.1186/s13104-021-05461-7 DOI for this manual: 10.17632/hfynpvtrz3

Mapping Electron Density

- Select **File** → Select **Open** → Open the given checkpoint file, **CH2O.fchk**

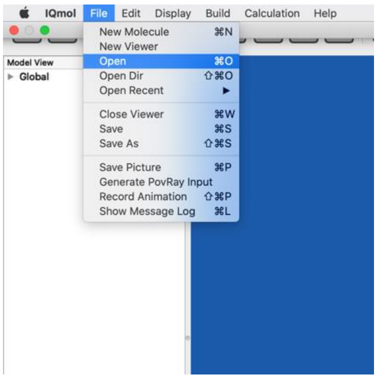

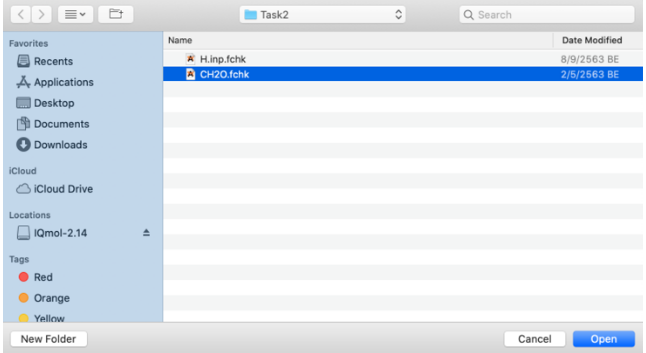

- In the **Model** View → double click **Surfaces** → **Calculate**

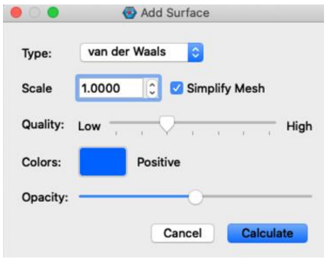

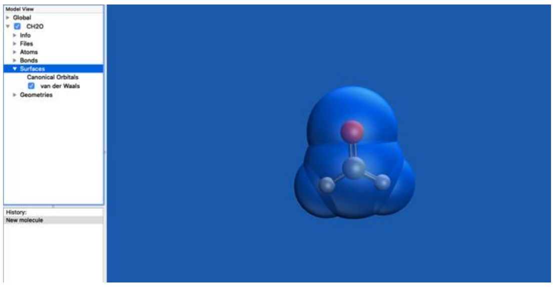

Generating Molecular Orbitals (HOMO and LUMO)

HOMO

- Under **Surfaces** → Double click **Canonical Orbitals** → Calculate → Unselect the **van der Waals** checkbox to hide the surface previously created

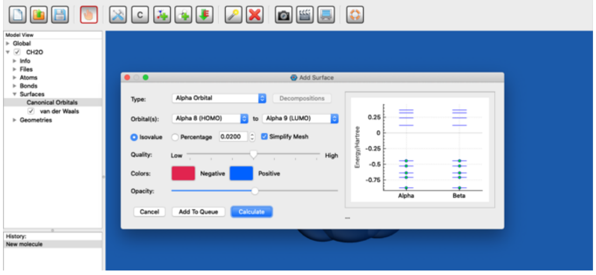

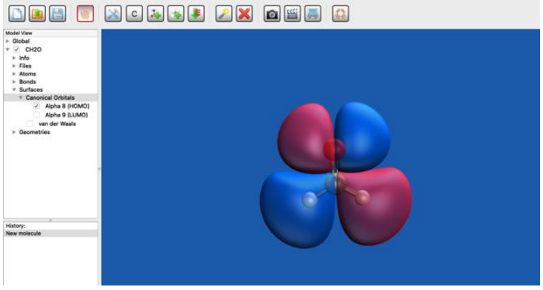

LUMO

Under **Canonical** Orbitals, unselect '**Alpha 8 (HOMO**)' checkbox and select **'Alpha 9 (LUMO)**'

DOI for the main paper: 10.1186/s13104-021-05461-7 DOI for this manual: 10.17632/hfynpvtrz3

Task 3: Predicting polarity of molecules

Generating Electrostatic Potential Map and Displaying Dipole-moment

- Build a H_2O molecule by selecting **Build element** icon 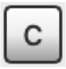 to choose an element wished to build. Click on an atom and drag to form a single bond. (Note: repeat the same step to increase the bond order) → Select **Minimize energy** icon 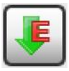

- In the **Model View**, double click **Surfaces** → **Calculate**

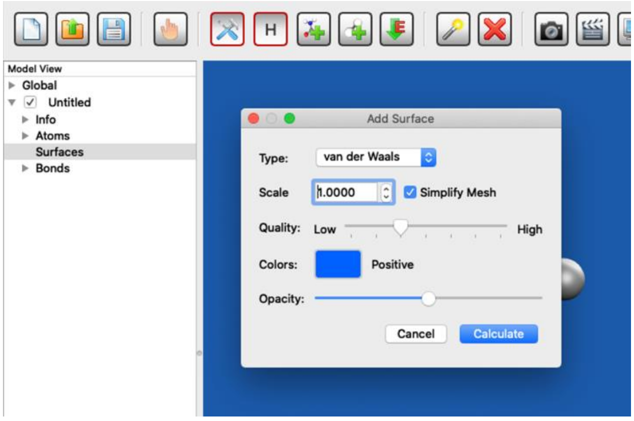

- Under **Surfaces**, double click **van der Waals** → For **Property**, select **ESP (Gasteiger)** → **OK**

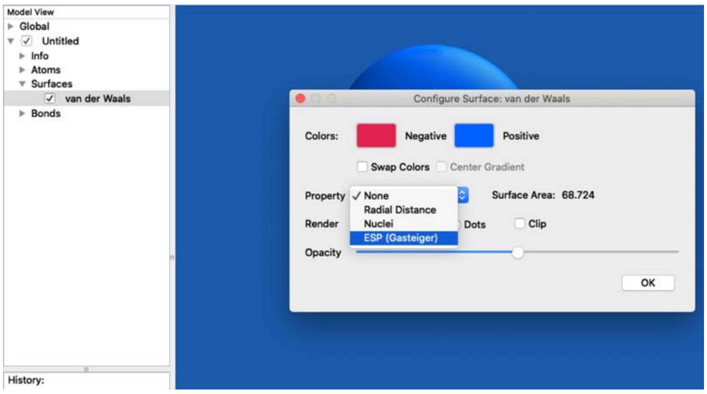

- To display a dipole-moment vector, under **Info**, tick the box to select **Dipole**.

If dipole-moment vector does not appear, you may need to click '**Minimize Energy'** button once (or twice)

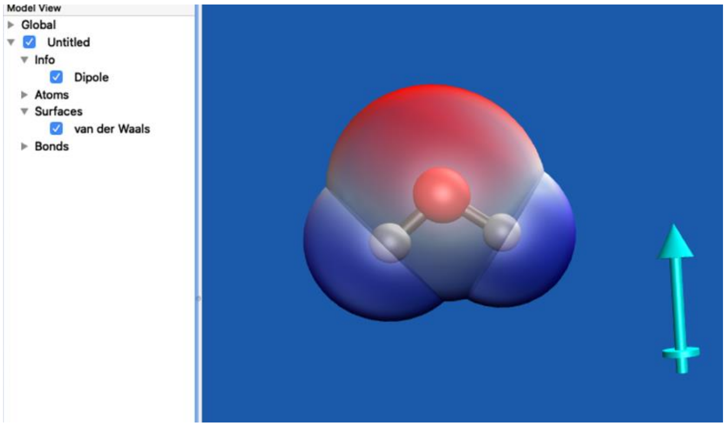

DOI for the main paper: 10.1186/s13104-021-05461-7 DOI for this manual: 10.17632/hfynpvtrz3

- To display the partial charge on each atom, select **Display → Atom Labels → Partial Charge**

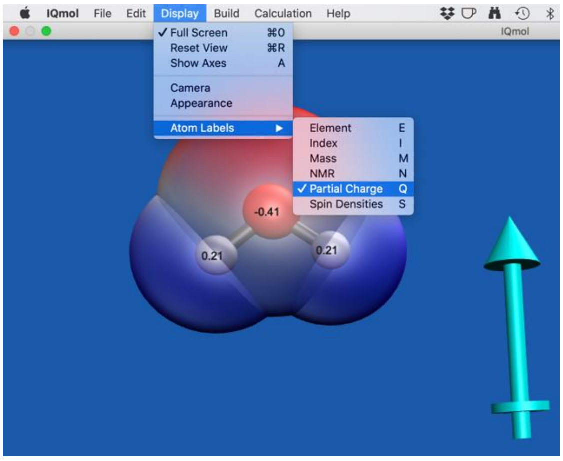

DOI for the main paper: 10.1186/s13104-021-05461-7 DOI for this manual: 10.17632/hfynpvtrz3

Task 4: Matching 3D structures with bond-line structures

- Select **File** → Select **Open**

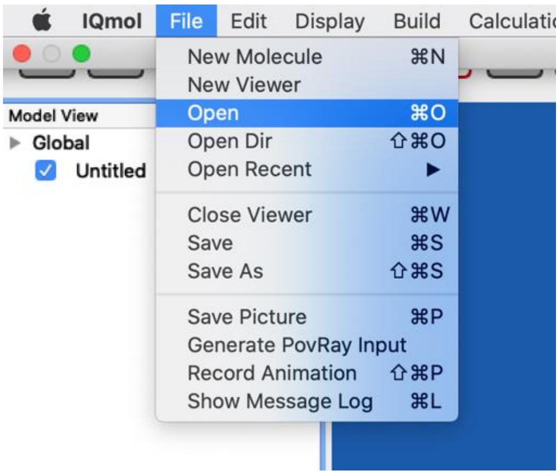

- Open each of the files in folder, **Task4** to display the 3D structure of each molecule

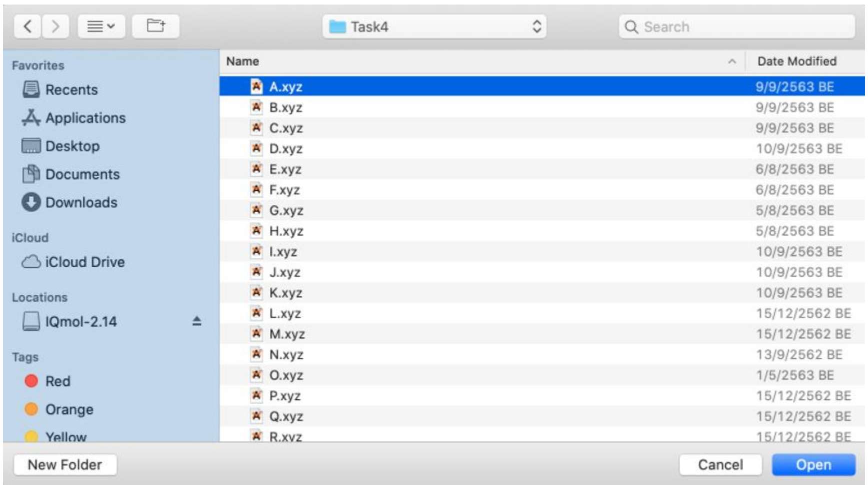

- Select **Manipulate** mode icon 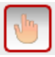 in the tool bar to adjust the perspective of the molecule

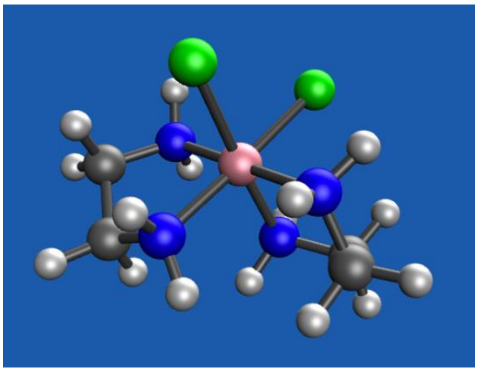

DOI for the main paper: 10.1186/s13104-021-05461-7 DOI for this manual: 10.17632/hfynpvtrz3
