# Supplementary material for: A virtual alternative to molecular model sets: a beginners’ guide to constructing and visualizing molecules in open-source molecular graphics software
Source: BMC Res Notes. 2021 Feb 17;14:66. doi: 10.1186/s13104-021-05461-7 (PMC7887714; doi:10.1186/s13104-021-05461-7)
Supplement: Supplementary file 1 — Additional file 1. Worksheet and files for students. [file 13104_2021_5461_MOESM1_ESM.zip › Tasks_for_students/molecular_model_worksheet.docx]

**Section_____ Date_______________ Preferred Name_____________________ Student ID_____________ Seat No. ______**

**1. Building basic molecular geometries**: Build these molecules: CO_2_, BCl_3_, SO_2_, CH_4_, NH_3_, H_2_O, PCl_5_, SF_4_, ClF_3_, SF_6_, BrF_5_, XeF_4_ in the program. Coordinate files for molecule on left column are available for use as templates. Put pictures of molecules in the table below.

| Steric number (hybridization and bond angle) | Lone pairs | | |
| --- | --- | --- | --- |
|  | 0 | 1 | 2 |
| 2  (sp 180°) | (Linear) |  |  |
| 3  (sp^2^ 120°) | (Trigonal planar) | (Bent) |  |
| 4  (sp^3^ ~109.5°) | (Tetrahedral) | (Trigonal pyramidal) | (Bent_)_ |
| 5  (dsp^3^ 90°/120°) | (Trigonal bipyramidal) | (Seesaw) | (T-shaped) |
| 6  (d^2^sp^3^ 90°) | (Octahedral) | (Square pyramidal) | (Square planar) |

(1) VSEPR predicts the trend lp-lp > lp-bp > bp-bp for repulsions where lp=lone pair/bp=bond pair.
(2) For steric number > 4, d orbitals are involved so the number of electrons can exceed the octet rule in hypervalent molecules.

**2. Visualizing orbitals & densities**

Given a checkpoint file, construct atomic orbitals of a H atom for the 6 subshells (14 orbitals) shown below.

| 1s |  |  |
| --- | --- | --- |
| 2s | 2p |  |
| 3s | 3p | 3d  (circle $\text{d}_{z^{2}}$) |

Given a checkpoint file, construct a total electron density map, HOMO and LUMO of formaldehyde. Use the default isovalue.

| Total electron density map | HOMO stands for .............................................. | LUMO stands for .............................................. |
| --- | --- | --- |
|  |  |  |

**3. Predicting polarity of molecules**

Show an electrostatic potential map (blue for positive and red for negative) and a dipole moment vector (pointing towards the negative end of the structure) for each of molecules listed below by using default molecular mechanics of the program. Does the picture show a polar bond/polar molecule? (Put two answers in the two blanks below the picture respectively.)

| H_2_O | | CO_2_ | | CH_4_ | | OF_2_ | | H_2_ | |
| --- | --- | --- | --- | --- | --- | --- | --- | --- | --- |
|  | |  | |  | |  | |  | |
|  |  |  |  |  |  |  |  |  |  |

Show dipole moment of ozone built and optimized by the program and a supplied ozone file. Is ozone a polar molecule? Why?

**4. Matching 3D structures with bond-line structures**

Inspect given files and put the file name (one letter alphabet) to the corresponding structure below. Write **‘no solution’** if a match cannot be found.

| **Wedge-and-dash projection** | | | **Fischer projection** | **Newman projection** | **Haworth projection** |
| --- | --- | --- | --- | --- | --- |
|  |  |  |  |  |  |
|  |  |  |  |  |  |

| **Bond-line structures** | | | | | |
| --- | --- | --- | --- | --- | --- |
|  |  |  |  |  |  |
|  |  |  |  |  |  |
