# Supplementary material for: A virtual alternative to molecular model sets: a beginners’ guide to constructing and visualizing molecules in open-source molecular graphics software
Source: BMC Res Notes. 2021 Feb 17;14:66. doi: 10.1186/s13104-021-05461-7 (PMC7887714; doi:10.1186/s13104-021-05461-7)
Supplement: Supplementary file 1 — Additional file 1. Worksheet and files for students. [file 13104_2021_5461_MOESM1_ESM.zip › Tasks_for_students/molecular_model_worksheet.pdf]

Section_____ Date_______________ Preferred Name_____________________ Student ID_____________ Seat No. ______

1. Building basic molecular geometries: Build these molecules: CO_2, BCl_3, SO_2, CH_4, NH_3, H_2O, PCl_5, SF_4, ClF_3, SF_6, BrF_5, XeF_4 in the program. Coordinate files for molecule on left column are available for use as templates. Put pictures of molecules in the table below.

Steric number (hybridization and bond angle)	Lone pairs		
	0	1	2
2 (sp 180°)	(Linear)		
3 (sp^2 120°)	(Trigonal planar)	(Bent)	
4 (sp^3 ~109.5°)	(Tetrahedral)	(Trigonal pyramidal)	(Bent)
5 (dsp^3 90°/120°)	(Trigonal bipyramidal)	(Seesaw)	(T-shaped)
6 (d^2sp^3 90°)	(Octahedral)	(Square pyramidal)	(Square planar)

(1) VSEPR predicts the trend lp-lp > lp-bp > bp-bp for repulsions where lp=lone pair/bp=bond pair.
(2) For steric number > 4, d orbitals are involved so the number of electrons can exceed the octet rule in hypervalent molecules.

2. Visualizing orbitals & densities

Given a checkpoint file, construct atomic orbitals of a H atom for the 6 subshells (14 orbitals) shown below.

1s		
2s	2p	
3s	3p	3d (circle d_{z^2})

Given a checkpoint file, construct a total electron density map, HOMO and LUMO of formaldehyde. Use the default isovalue.

Total electron density map	HOMO stands for	LUMO stands for ....

3. Predicting polarity of molecules

Show an electrostatic potential map (blue for positive and red for negative) and a dipole moment vector (pointing towards the negative end of the structure) for each of molecules listed below by using default molecular mechanics of the program. Does the picture show a polar bond/polar molecule? (Put two answers in the two blanks below the picture respectively.)

H_2O		CO_2		CH_4		OF_2		H_2	

Show dipole moment of ozone built and optimized by the program and a supplied ozone file. Is ozone a polar molecule? Why?

4. Matching 3D structures with bond-line structures

Inspect given files and put the file name (one letter alphabet) to the corresponding structure below. Write **'no solution'** if a match cannot be found.

**Wedge-and-dash projection**			**Fischer projection**	**Newman projection**	**Haworth projection**
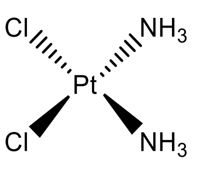	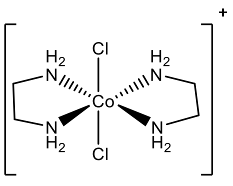	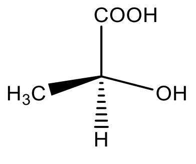	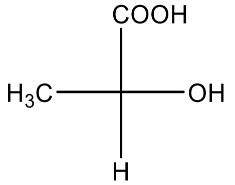	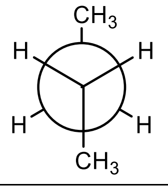	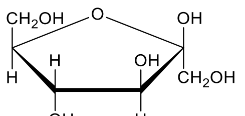

**Bond-line structures**					
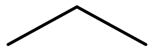	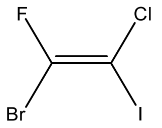	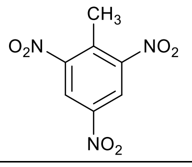	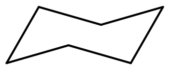	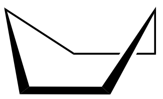	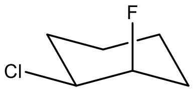

DOI: 10.1186/s13104-021-05461-7
