# Supplementary material for: A virtual alternative to molecular model sets: a beginners’ guide to constructing and visualizing molecules in open-source molecular graphics software
Source: BMC Res Notes. 2021 Feb 17;14:66. doi: 10.1186/s13104-021-05461-7 (PMC7887714; doi:10.1186/s13104-021-05461-7)
Supplement: Supplementary file 2 — Additional file 2. Grading criteria and complete solutions inclusive of optional exercises. [file 13104_2021_5461_MOESM2_ESM.zip › Grading_rubric.docx]

**Grading Rubric**

**Task 1**

| **Criteria** | **Full mark** | **Student score** |
| --- | --- | --- |
| Molecular geometries are correctly shown. | 7 |  |
| **Subtotal** | 7 |  |

**Task 2**

| **Criteria** | **Full mark** | **Student score** |
| --- | --- | --- |
| Atomic orbitals are correctly labelled. | 3 |  |
| Electron density map is correctly constructed. | 1 |  |
| Molecular orbitals (HOMO and LUMO) are correctly described. | 3 |  |
| **Subtotal** | 7 |  |

**Task 3**

| **Criteria** | **Full mark** | **Student score** |
| --- | --- | --- |
| Electrostatic potential maps are correctly constructed for the molecules assigned. | 3 |  |
| Dipole moments are correctly presented for the molecules assigned. | 2 |  |
| Questions regarding polarity are correctly answered. | 3 |  |
| **Subtotal** | 8 |  |

**Task 4**

| **Criteria** | **Full mark** | **Student score** |
| --- | --- | --- |
| 3D molecular models are correctly assigned to 2D representations. | 8 |  |
| **Subtotal** | 8 |  |

|  | **Full mark** | **Student score** |
| --- | --- | --- |
| Task 1 | 7 |  |
| Task 2 | 7 |  |
| Task 3 | 8 |  |
| Task 4 | 8 |  |
| **Total** | **30** |  |
