# Supplementary material for: A virtual alternative to molecular model sets: a beginners’ guide to constructing and visualizing molecules in open-source molecular graphics software
Source: BMC Res Notes. 2021 Feb 17;14:66. doi: 10.1186/s13104-021-05461-7 (PMC7887714; doi:10.1186/s13104-021-05461-7)
Supplement: Supplementary file 2 — Additional file 2. Grading criteria and complete solutions inclusive of optional exercises. [file 13104_2021_5461_MOESM2_ESM.zip › Grading_rubric.pdf]

Grading Rubric

Task 1

Criteria	Full mark	Student score
Molecular geometries are correctly shown.	7	
**Subtotal**	7	

Task 2

Criteria	Full mark	Student score
Atomic orbitals are correctly labelled.	3	
Electron density map is correctly constructed.	1	
Molecular orbitals (HOMO and LUMO) are correctly described.	3	
**Subtotal**	7	

Task 3

Criteria	Full mark	Student score
Electrostatic potential maps are correctly constructed for the molecules assigned.	3	
Dipole moments are correctly presented for the molecules assigned.	2	
Questions regarding polarity are correctly answered.	3	
**Subtotal**	8	

Task 4

Criteria	Full mark	Student score
3D molecular models are correctly assigned to 2D representations.	8	
**Subtotal**	8	

	Full mark	Student score
Task 1	7	
Task 2	7	
Task 3	8	
Task 4	8	
**Total**	**30**	

DOI: 10.1186/s13104-021-05461-7
