# Supplementary material for: A virtual alternative to molecular model sets: a beginners’ guide to constructing and visualizing molecules in open-source molecular graphics software
Source: BMC Res Notes. 2021 Feb 17;14:66. doi: 10.1186/s13104-021-05461-7 (PMC7887714; doi:10.1186/s13104-021-05461-7)

**1. Building basic molecular geometries**

Build these molecules: CO_2_, BCl_3_, SO_2_, CH_4_, NH_3_, H_2_O, PCl_5_, SF_4_, ClF_3_, SF_6_, BrF_5_, XeF_4_ in the program. Coordinate files for molecule on left column are available for use as templates. Put pictures of molecules in the table below.

| Steric number (hybridization and bond angle) | Lone pairs | | |
| --- | --- | --- | --- |
|  | 0 | 1 | 2 |
| 2  (sp 180°) | 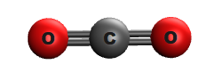(Linear) |  |  |
| 3  (sp^2^ 120°) | 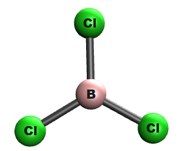 (Trigonal planar) | 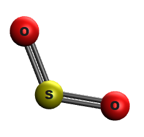 (Bent) |  |
| 4  (sp^3^ ~109.5°) | 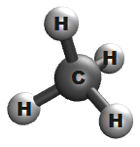 (Tetrahedral) | 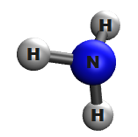 (Trigonal pyramidal) | 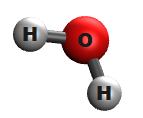 (Bent) |
| 5  (dsp^3^ 90°/120°) | 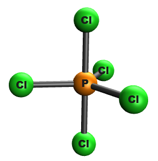 (Trigonal bipyramidal) | 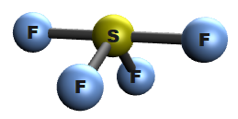(Seesaw) | 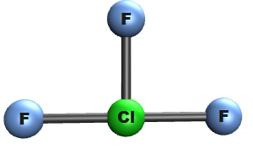  (T-shaped) |
| 6  (d^2^sp^3^ 90°) | 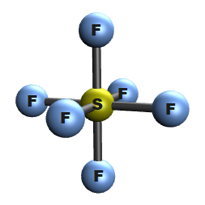(Octahedral) | 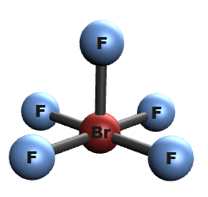(Square pyramidal) | 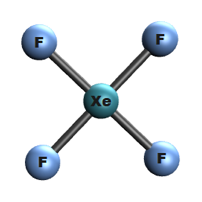 (Square planar) |

(1) VSEPR predicts the trend lp-lp > lp-bp > bp-bp for repulsions where lp=lone pair/bp=bond pair.
(2) For steric number > 4, d orbitals are involved so the number of electrons can exceed the octet rule in hypervalent molecules.

- Where does the bond angles of 180°, 120°, ~109.5° and 90° come from? Majority of these come from 360°/*n*. For tetrahedral, the exact value is $\cos^{-1} \left( -\frac{1}{3} \right)$ which can be derived from the dot product of vectors if hydrogens are placed at four out of eight corners of a cube.
- Predict the shape of XeF_2_ and its position in the table above. This molecule is linear and should be to right of ClF_3_.
- Show the structure of hydrogen halides in space filling model.


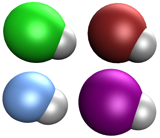


- Use forcefield energy minimization or published geometries to explore bond lengths and bond angles in water, methane and fluoromethanes (10.1016/j.dib.2020.105442). Use VSEPR/Bent’s rule or other advanced theory (10.1021/jp502472u) to rationalize your observations.
  - UFF’s Bond angles: Methane ~109.5°, ammonia ~109.2°, water ~104.5°. The bond pair orbitals are having more p character from methane to water. As a result, the bond angle decreases accordingly.
  - The discussion of Bent’s rule and its extensions can be found in textbooks and given references. A more advanced discussion is beyond the scope of this brief introductory exercise to molecular modelling.

**2. Visualizing orbitals and densities**

Given a checkpoint file, construct atomic orbitals of a H atom for the 6 subshells (14 orbitals) shown below.

| 1s  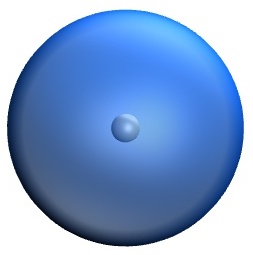 |  |  |
| --- | --- | --- |
| 2s  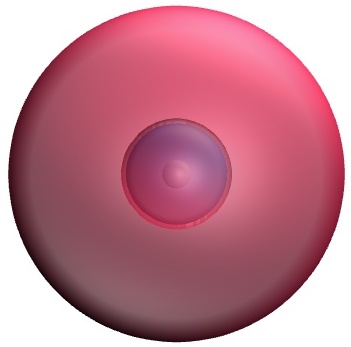 | 2p  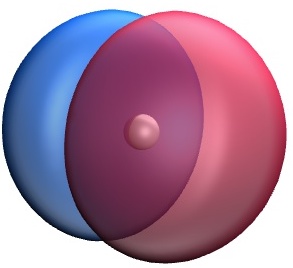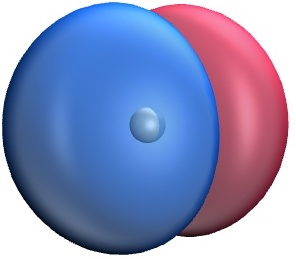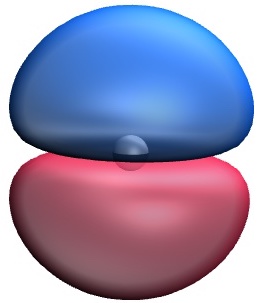 |  |
| 3s  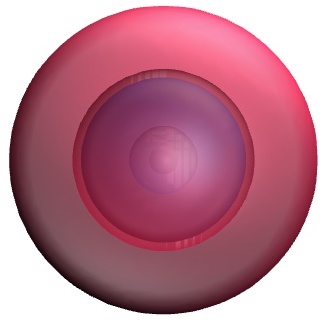 | 3p  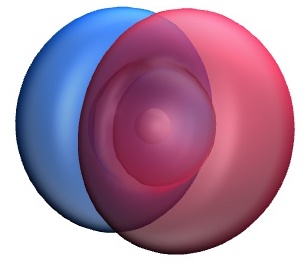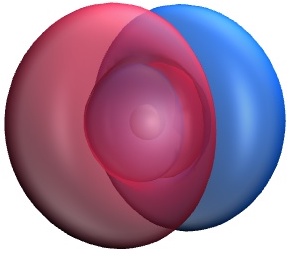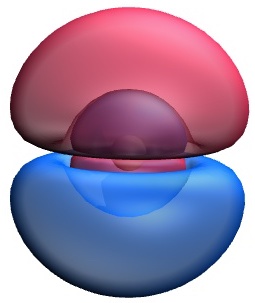 | 3d  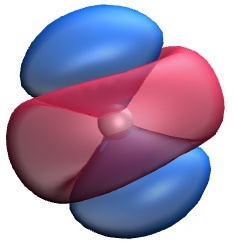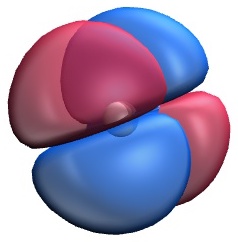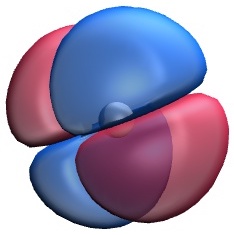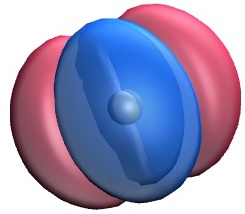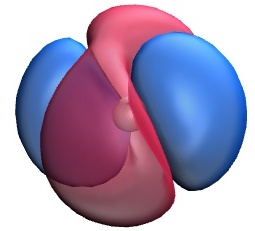  (circle $\text{d}_{z^{2}}$) |

Given a checkpoint file, construct three electron density maps of formaldehyde for three different isovalues.

Construct HOMO and LUMO for a formaldehyde molecule and type full forms for the two acronyms.

| Total electron density map | | | HOMO stands for Highest Occupied Molecular Orbital | LUMO stands for Lowest Unoccupied Molecular Orbital |
| --- | --- | --- | --- | --- |
| Isovalue = 0.0020 | Isovalue = 0.0200  (default value) | Isovalue = 2.000 |  |  |
| 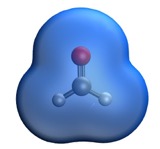 | 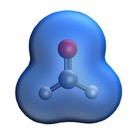 | 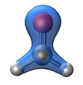 | 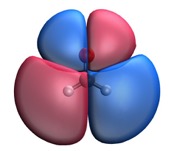 | 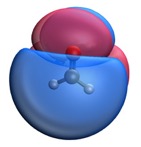 |

**3. Predicting polarity of molecules**

Show an electrostatic potential map (blue for positive and red for negative) and a dipole moment vector (pointing towards the negative end of the structure) for each of molecules listed below by using default molecular mechanics of the program. Does the picture show a polar bond/polar molecule? (Put two answers in the two blanks below the picture respectively.)

| H_2_O | | CO_2_ | | CH_4_ | | OF_2_ | | H_2_ | |
| --- | --- | --- | --- | --- | --- | --- | --- | --- | --- |
| 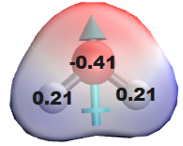 | | 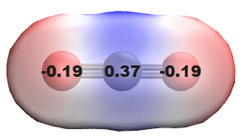 | | 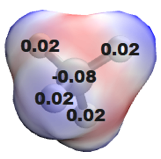 | | 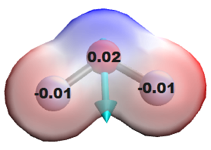 | | 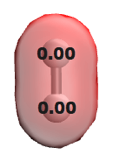 | |
| Yes | Yes | Yes | No | Yes (weakly) | No | Yes | Yes | No | No |

Show dipole moment of ozone built and optimized by the program and a supplied ozone file. Is ozone a polar molecule? Why?

| Ozone built and optimized by internal molecular mechanics of the software | Ozone from a quantum chemical calculation  (O3.out is supplied to students.) | Resonance structure in ozone |
| --- | --- | --- |
| 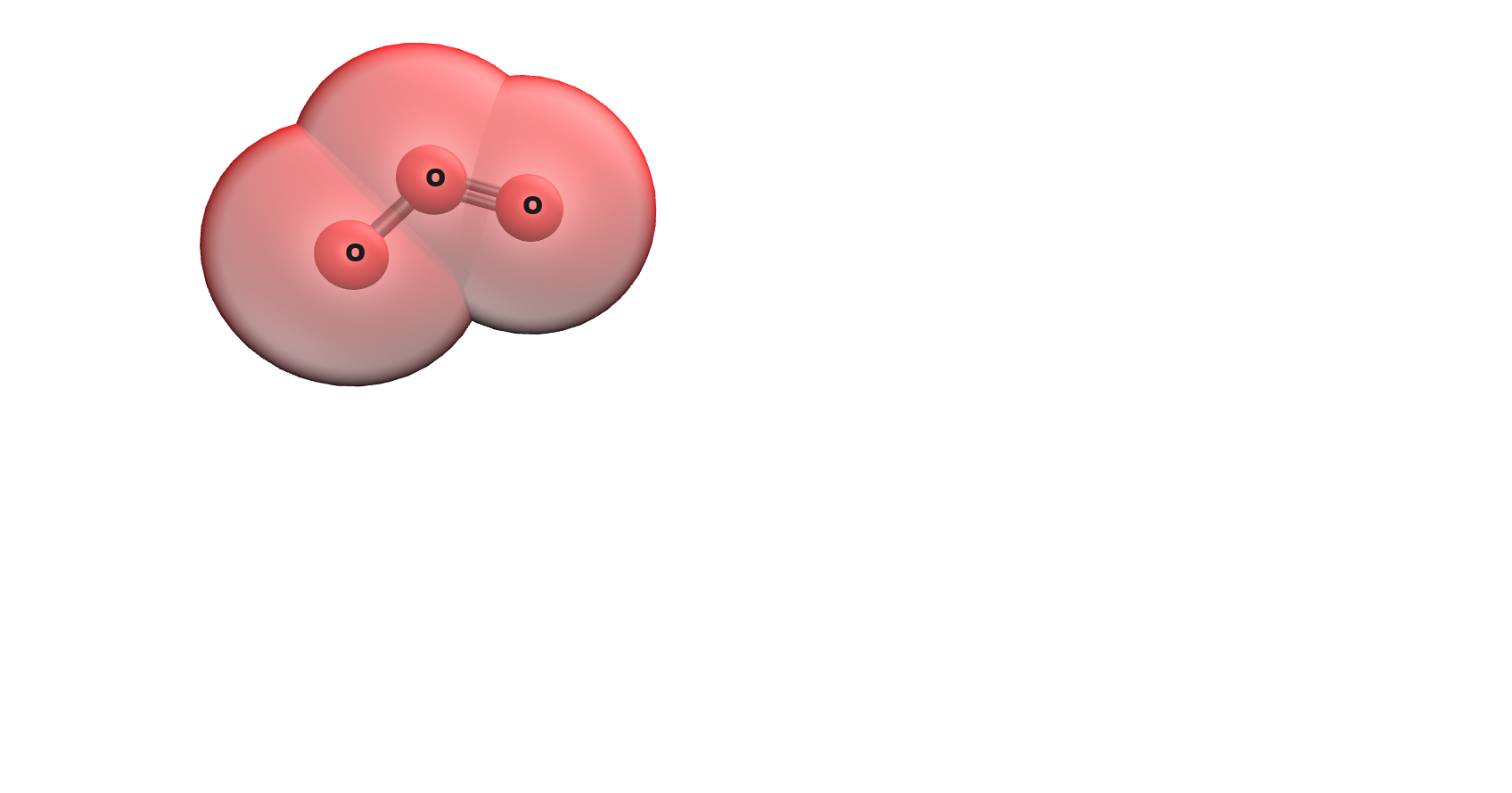 | 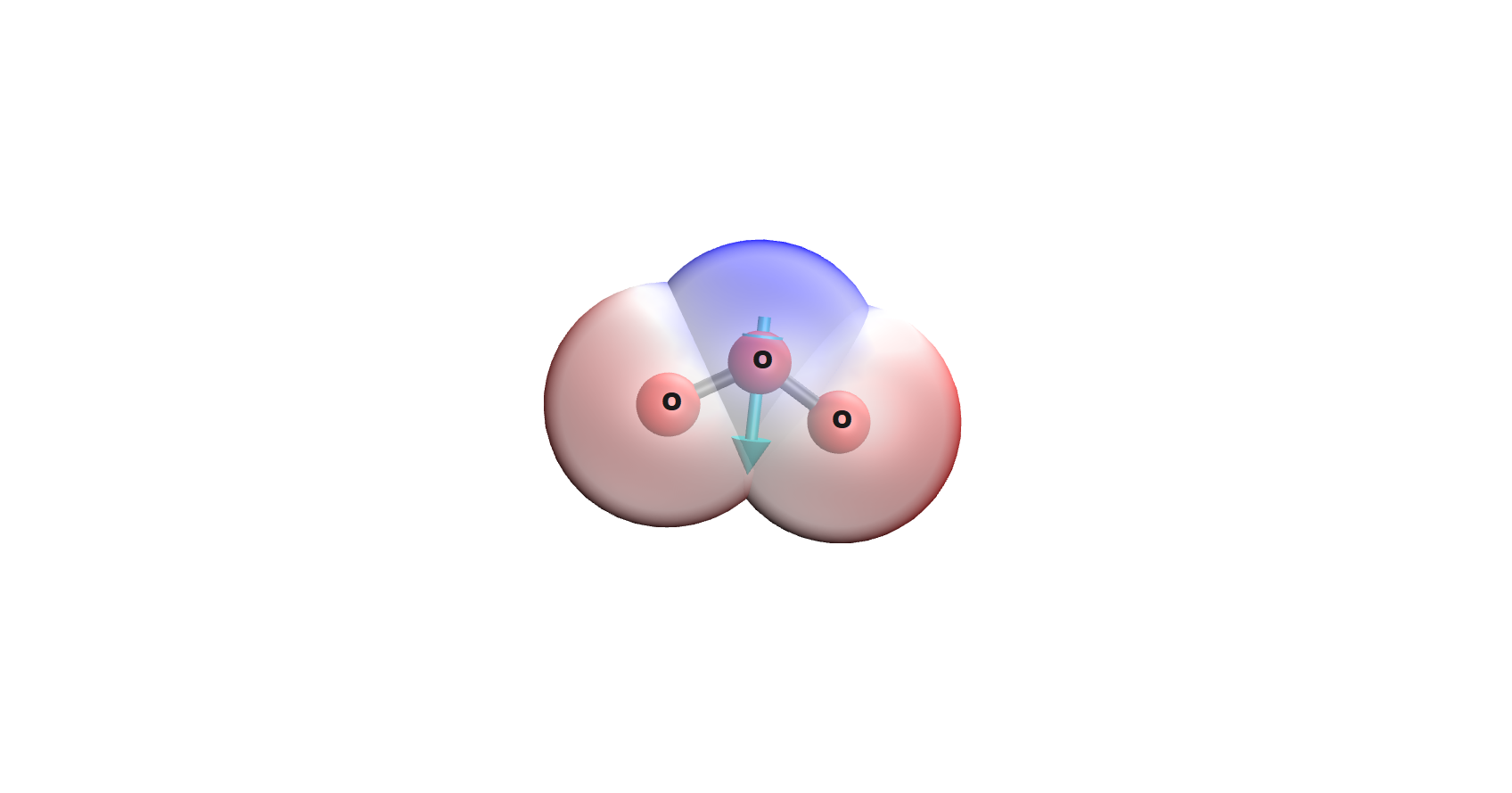 |  |

- Yes, it is. The bent structure and formal charge on atoms make it polar. This is correctly illustrated by a net dipole moment pointing down in the figure above. Due to unequal distribution of charges, the two outer oxygen atoms more negative than the central oxygen. This is correctly shown by a supplied ozone file. Although there are no electronegativity differences, the bond dipole moment can arise. The dipole moment of the molecule is the vector sum of the bond dipole moment. Students should understand limitation of molecular mechanics and appreciate the needs for quantum chemical calculations.

Show the IR spectrum of acetic acid (with Gaussian fitting) and the vibrational mode corresponding to the highest frequency


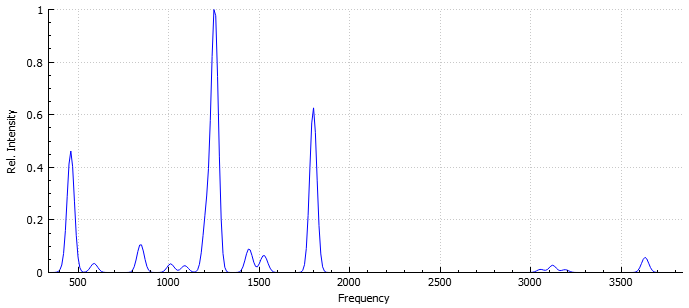
**
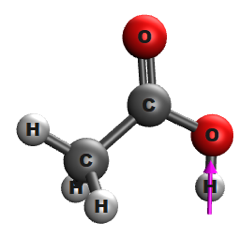
**

OH stretching has the highest frequency.

**4. Matching 3D structures with bond-line structures**

Inspect given files and put the file name (one letter alphabet) to the corresponding structure below. Write **‘no solution’** if a match cannot be found.

| **Wedge-and-dash projection** | | | **Fischer**  **projection** | **Newman projection** | **Haworth projection** |
| --- | --- | --- | --- | --- | --- |
|  |  | **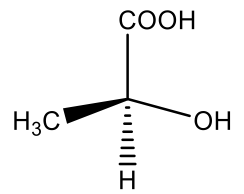** | **** | **** | **** |
| H | C | M | M | R | No solution |

| **Bond-line structure** | | | | | |
| --- | --- | --- | --- | --- | --- |
|  |  |  |  |  |  |
| I | V | S | N | O | Y |

From the structures listed above, identify all isomers/rotamers. If possible, identify which isomer is more stable.

- Gauche, anti, eclipsed are rotamers. Stability: Anti > Gauche > Eclipsed
- Chair and boat are rotamers. Stability: Chair > Boat
- 1-chloro-2-fluorocyclohexane are stereoisomers. The one with Cl on an equatorial position is likely to be more stable.

From the given structure files, identify all enantiomer pairs (exact mirror image or mirror image after single bond rotation)

- D-(-)-lactic acid and L-(+)-lactic acid
- (1*S*,2*R*)-1-chloro-2-fluorocyclohexane (F is equatorial) and (1*R*,2*S*)-1-chloro-2-fluorocyclohexane
- (1*S*,2*R*)-1-chloro-2-fluorocyclohexane (Cl is equatorial) and (1*R*,2*S*)-1-chloro-2-fluorocyclohexane (after rotation)
- (1*R*,2*R*)-1-chloro-2-fluorocyclohexane (halogens are axial) and (1*S*,2*S*)-1-chloro-2-fluorocyclohexane (halogens are equatorial) (after rotation)

Use the program to convert a chair form of (1*S*,2*R*)-1-chloro-2-fluorocyclohexane (Cl is equatorial) to the other chair form where Cl is axial.

Research for the chemical structure of common molecules found in your everyday life and draw it yourself using the software.

Structures of acetaminophen (paracetamol) and acetylsalicylic acid (aspirin) are shown below as an example.


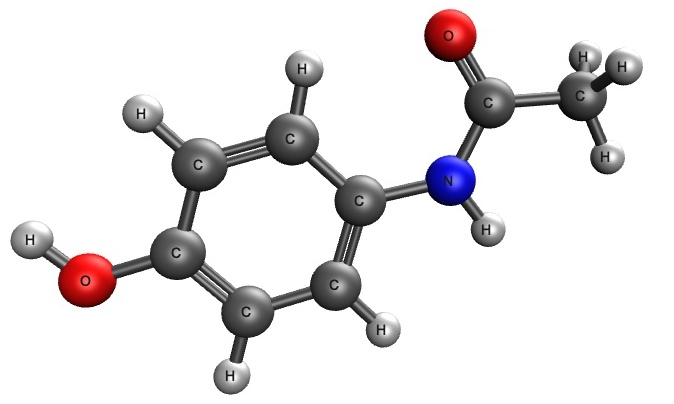

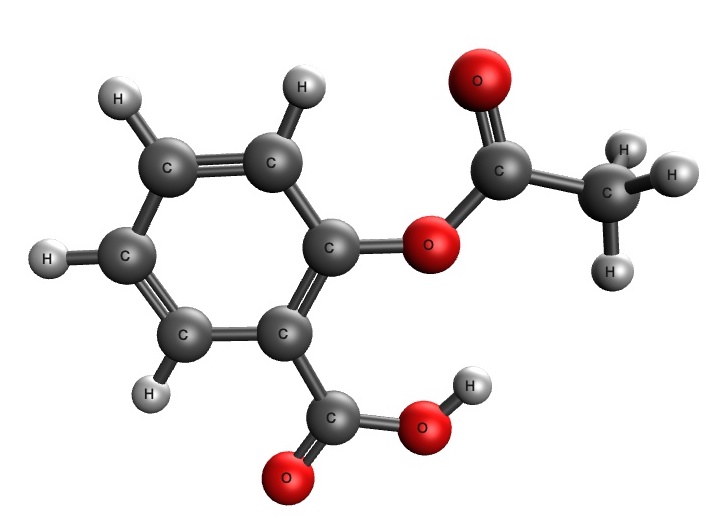

Supplement: Supplementary file 2 — Additional file 2. Grading criteria and complete solutions inclusive of optional exercises. [file 13104_2021_5461_MOESM2_ESM.zip › Solution_IQmol.docx]
