# Supplementary material for: A virtual alternative to molecular model sets: a beginners’ guide to constructing and visualizing molecules in open-source molecular graphics software
Source: BMC Res Notes. 2021 Feb 17;14:66. doi: 10.1186/s13104-021-05461-7 (PMC7887714; doi:10.1186/s13104-021-05461-7)
Supplement: Supplementary file 2 — Additional file 2. Grading criteria and complete solutions inclusive of optional exercises. [file 13104_2021_5461_MOESM2_ESM.zip › Task4/List of structures.docx]

| **File name** | **Name** | **Bond-line struture** |
| --- | --- | --- |
| A | Λ-*cis*-dichlorobis(ethylenediamine)cobalt(III) ion |  |
| B | Δ-*cis*-dichlorobis(ethylenediamine)cobalt(III) ion |  |
| C | *trans*-dichlorobis(ethylenediamine)cobalt(III) ion | ** |
| D | β -L-glucopyranose |  |
| E | β-D-fructofuranose |  |
| F | α-D-fructofuranose |  |
| G | *trans*-platin | ** |
| H | *cis*-platin | ** |
| I | propane |  |
| J | propa-1,2-diene |  |
| K | prop-1-ene |  |
| L | l-(+)-lactic acid |  |
| M | d-(-)-lactic acid |  |
| N | cyclohexane_chair_form |  |
| O | cyclohexane_boat_form |  |
| P | butane_gauche_conformer |  |
| Q | butane_eclipsed_conformation |  |
| R | butane_anti_conformer |  |
| S | 2-methyl-1,3,5-trinitrobenzene (TNT) |  |
| T | 1,2-dimethyl-3,5-dinitrobenzene |  |
| U | 1-methyl-2,4-dinitrobenzene |  |
| V | (*Z*)-1-bromo-2-chloro-1-fluoro-2-iodoethene |  |
| W | (*E*)-1-bromo-2-chloro-1-fluoro-2-iodoethene |  |
| X | (1*S*,2*R*)-1-chloro-2-fluorocyclohexane (F is equatorial) |  |
| Y | (1*S*,2*R*)-1-chloro-2-fluorocyclohexane (Cl is equatorial) |  |
| Z | (1*R*,2*R*)-1-chloro-2-fluorocyclohexane (halogens are axial) |  |
