# Supplementary material for: A virtual alternative to molecular model sets: a beginners’ guide to constructing and visualizing molecules in open-source molecular graphics software
Source: BMC Res Notes. 2021 Feb 17;14:66. doi: 10.1186/s13104-021-05461-7 (PMC7887714; doi:10.1186/s13104-021-05461-7)
Supplement: Supplementary file 2 — Additional file 2. Grading criteria and complete solutions inclusive of optional exercises. [file 13104_2021_5461_MOESM2_ESM.zip › Task4/List of structures.pdf]

File name	Name	Bond-line struture
A	Λ-*cis*-dichlorobis(ethylenediamine)cobalt(III) ion	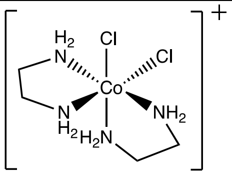
B	Δ-*cis*-dichlorobis(ethylenediamine)cobalt(III) ion	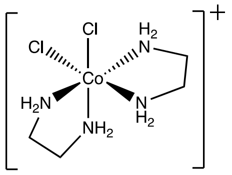
C	*trans*-dichlorobis(ethylenediamine)cobalt(III) ion	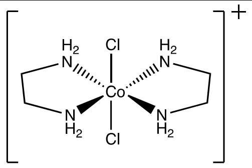
D	β -L-glucopyranose	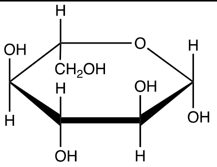
E	β-D-fructofuranose	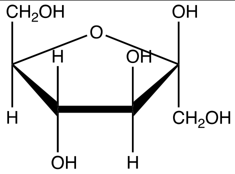
F	α-D-fructofuranose	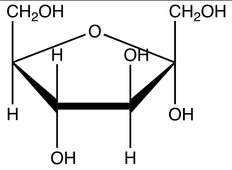
G	*trans*-platin	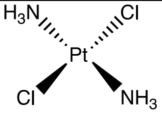
H	*cis*-platin	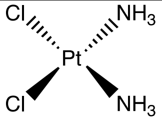
I	propane	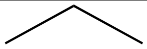
J	propa-1,2-diene	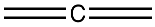
K	prop-1-ene	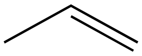
L	L-(+)-lactic acid	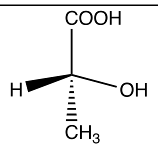
M	D-(-)-lactic acid	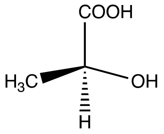
N	cyclohexane_chair_form	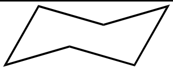
O	cyclohexane_boat_form	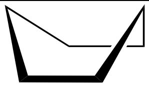

DOI: 10.1186/s13104-021-05461-7

P	butane_gauche_conformer	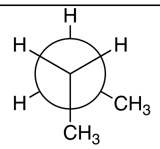
Q	butane_eclipsed_conformation	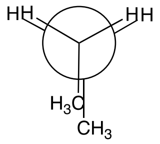
R	butane_anti_conformer	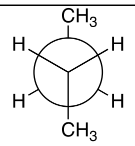
S	2-methyl-1,3,5-trinitrobenzene (TNT)	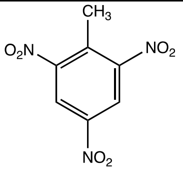
T	1,2-dimethyl-3,5-dinitrobenzene	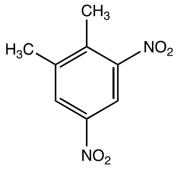
U	1-methyl-2,4-dinitrobenzene	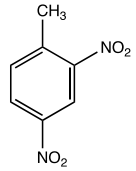
V	(*Z*)-1-bromo-2-chloro-1-fluoro-2-iodoethene	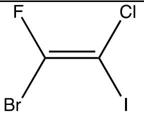
W	(*E*)-1-bromo-2-chloro-1-fluoro-2-iodoethene	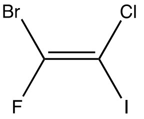
X	(1*S*,2*R*)-1-chloro-2-fluorocyclohexane (F is equatorial)	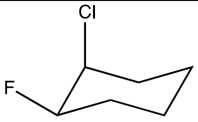
Y	(1*S*,2*R*)-1-chloro-2-fluorocyclohexane (Cl is equatorial)	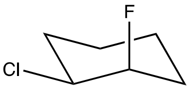
Z	(1*R*,2*R*)-1-chloro-2-fluorocyclohexane (halogens are axial)	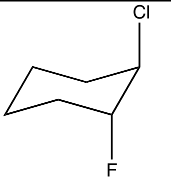

DOI: 10.1186/s13104-021-05461-7
