# Supplementary figures and images for: A virtual alternative to molecular model sets: a beginners’ guide to constructing and visualizing molecules in open-source molecular graphics software
Source: BMC Res Notes. 2021 Feb 17;14:66. doi: 10.1186/s13104-021-05461-7 (PMC7887714; doi:10.1186/s13104-021-05461-7)

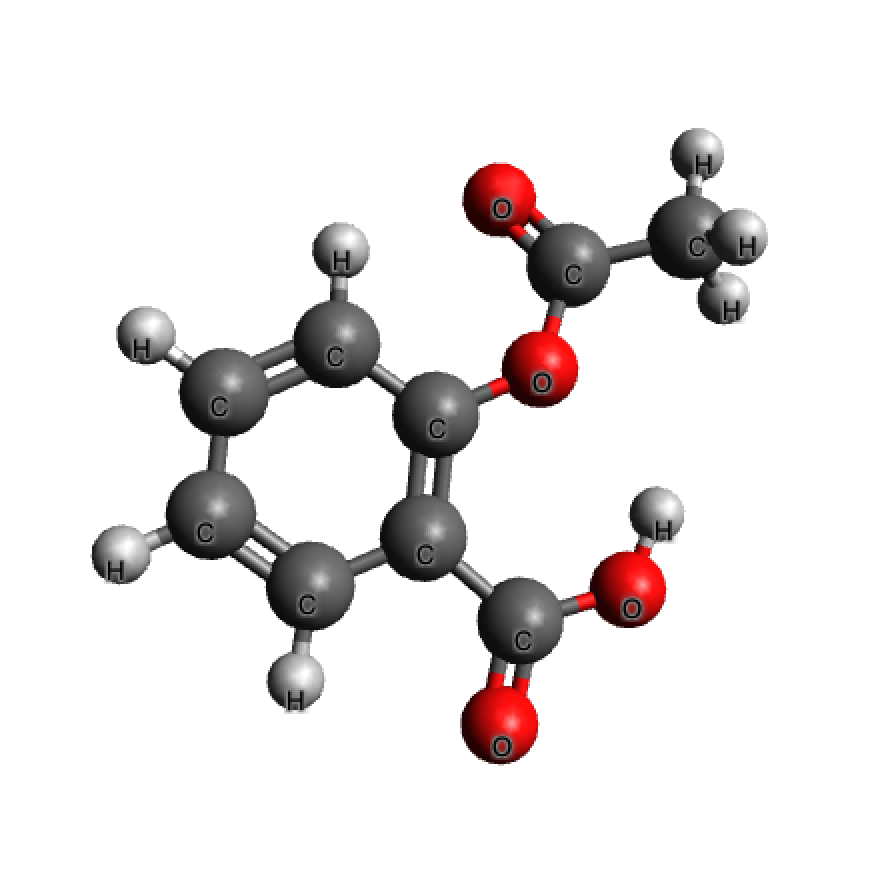

Supplement: Supplementary file 2 — Additional file 2. Grading criteria and complete solutions inclusive of optional exercises. [file 13104_2021_5461_MOESM2_ESM.zip › Task4/Aspirin_Avogadro.png]

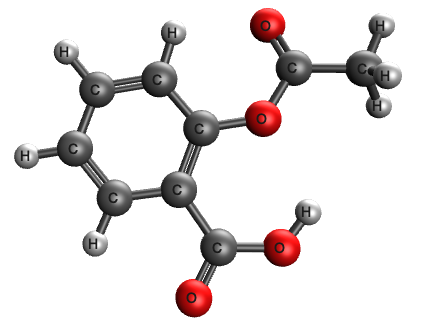

Supplement: Supplementary file 2 — Additional file 2. Grading criteria and complete solutions inclusive of optional exercises. [file 13104_2021_5461_MOESM2_ESM.zip › Task4/Aspirin_iqmol.png]

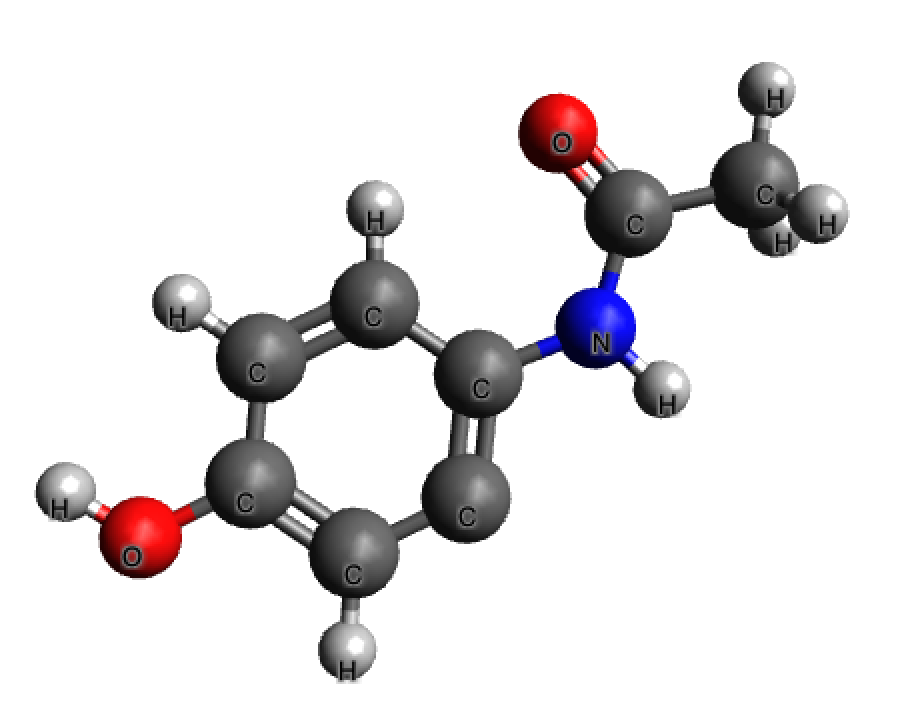

Supplement: Supplementary file 2 — Additional file 2. Grading criteria and complete solutions inclusive of optional exercises. [file 13104_2021_5461_MOESM2_ESM.zip › Task4/Paracetamol_Avogadro.png]

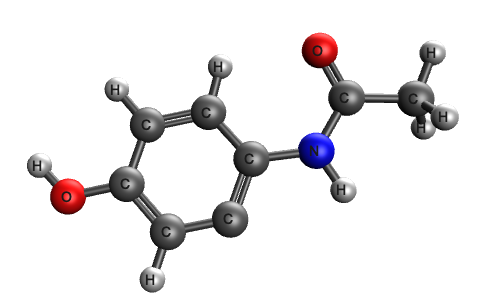

Supplement: Supplementary file 2 — Additional file 2. Grading criteria and complete solutions inclusive of optional exercises. [file 13104_2021_5461_MOESM2_ESM.zip › Task4/Paracetamol_iqmol.png]
