# Supplementary material for: A virtual alternative to molecular model sets: a beginners’ guide to constructing and visualizing molecules in open-source molecular graphics software
Source: BMC Res Notes. 2021 Feb 17;14:66. doi: 10.1186/s13104-021-05461-7 (PMC7887714; doi:10.1186/s13104-021-05461-7)
Supplement: Supplementary file 3 — Additional file 3. Survey questions and detailed results. [file 13104_2021_5461_MOESM3_ESM.zip › Survey/paired t-test/Jan2021.pdf]

```
T-TEST PAIRS=Pre WITH Post (PAIRED)
  /CRITERIA=CI(.9500)
  /MISSING=ANALYSIS.
```

T-Test

```
[DataSet0]
```

Paired Samples Statistics

		Mean	N	Std. Deviation	Std. Error Mean
Pair 1	Pre	.5761	23	.19121	.03987
	Post	.6848	23	.22885	.04772

Paired Samples Correlations

		N	Correlation	Sig.
Pair 1	Pre & Post	23	-.206	.346

Paired Samples Test

		Paired Differences				
					95% Confidence Interval of the Difference	
		Mean	Std. Deviation	Std. Error Mean	Lower	Upper
Pair 1	Pre - Post	-.10870	.32706	.06820	-.25013	.03273

Paired Samples Test

		t	df	Sig. (2-tailed)
Pair 1	Pre - Post	-1.594	22	.125
