# Supplementary material for: A virtual alternative to molecular model sets: a beginners’ guide to constructing and visualizing molecules in open-source molecular graphics software
Source: BMC Res Notes. 2021 Feb 17;14:66. doi: 10.1186/s13104-021-05461-7 (PMC7887714; doi:10.1186/s13104-021-05461-7)
Supplement: Supplementary file 3 — Additional file 3. Survey questions and detailed results. [file 13104_2021_5461_MOESM3_ESM.zip › Survey/paired t-test/Sep2020.pdf]

```
T-TEST PAIRS=pre WITH post (PAIRED)
  /CRITERIA=CI(.9500)
  /MISSING=ANALYSIS.
```

T-Test

Notes

Output Created		04-Oct-2020 16:48:59
Comments		
Input	Active Dataset	DataSet0
	Filter	<none>
	Weight	<none>
	Split File	<none>
	N of Rows in Working Data File	31
Missing Value Handling	Definition of Missing	User defined missing values are treated as missing.
	Cases Used	Statistics for each analysis are based on the cases with no missing or out-of-range data for any variable in the analysis.
Syntax		T-TEST PAIRS=pre WITH post (PAIRED) /CRITERIA=CI(.9500) /MISSING=ANALYSIS.
Resources	Processor Time	00:00:00.016
	Elapsed Time	00:00:00.152

```
[DataSet0]
```

Paired Samples Statistics

		Mean	N	Std. Deviation	Std. Error Mean
Pair 1	pre	.6290	31	.23158	.04159
	post	.7419	31	.19879	.03570

Paired Samples Correlations

		N	Correlation	Sig.
Pair 1	pre & post	31	-.067	.720

Paired Samples Test

		Paired Differences				
					95% Confidence Interval of the Difference	
		Mean	Std. Deviation	Std. Error Mean	Lower	Upper
Pair 1	pre - post	-.11290	.31516	.05661	-.22851	.00270

Paired Samples Test

		t	df	Sig. (2-tailed)
Pair 1	pre - post	-1.995	30	.055
