# Supplementary material for: A virtual alternative to molecular model sets: a beginners’ guide to constructing and visualizing molecules in open-source molecular graphics software
Source: BMC Res Notes. 2021 Feb 17;14:66. doi: 10.1186/s13104-021-05461-7 (PMC7887714; doi:10.1186/s13104-021-05461-7)
Supplement: Supplementary file 3 — Additional file 3. Survey questions and detailed results. [file 13104_2021_5461_MOESM3_ESM.zip › Survey/pre-and post tests.docx]

**SEC_____ No_____ Date ___________**

**These are pre-activity questions.**

**Please do not remove the staple until you are told to do so.**

1. What computer did you use for the activity today?

⃝ Your own computer ⃝ The university’s computer laboratory

2. What operating system did you use for the activity today?

⃝ Windows ⃝ Mac ⃝ Linux

3. A molecule with two lone pairs and two atoms bonded to the central atom has a bent structure.

⃝ True ⃝ False

4. The colours of atomic orbital in MOs correspond to their positive and negative charges.

⃝ True ⃝ False

5. Water is a polar molecule.

⃝ True ⃝ False

6. The molecular structure on the left is represented by the picture on the right.


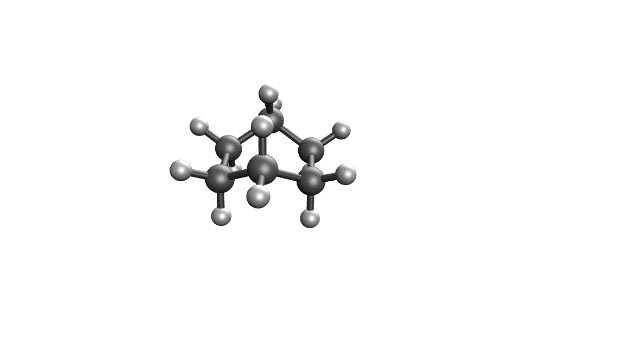


⃝ True ⃝ False

**These are post-activity questions.**

**The software**

| Avogadro | IQmol |
| --- | --- |
| 7a. Can you download the program?  ⃝ Yes ⃝ No, please specify reason(s) | 7b. Can you download the program?  ⃝ Yes ⃝ No, please specify reason(s) |
| 8a. Can you install the program?  ⃝ Yes ⃝ No, please specify reason(s) | 8b. Can you install the program?  ⃝ Yes ⃝ No, please specify reason(s) |
| 9a. Can you use the program to complete assigned tasks?  ⃝ Yes ⃝ No, please specify reason(s) | 9b. Can you use the program to complete assigned tasks?  ⃝ Yes ⃝ No, please specify reason(s) |

**Knowledge**

10. A molecule with zero lone pair and 6 atoms bonded to the central atom has an octahedral structure.

⃝ True ⃝ False

11. The colours of atomic orbital in MOs correspond to the phases of the wavefunction.

⃝ True ⃝ False

12. Methane is a polar molecule.

⃝ True ⃝ False

13. The molecular structure on the left is represented by the picture on the right.


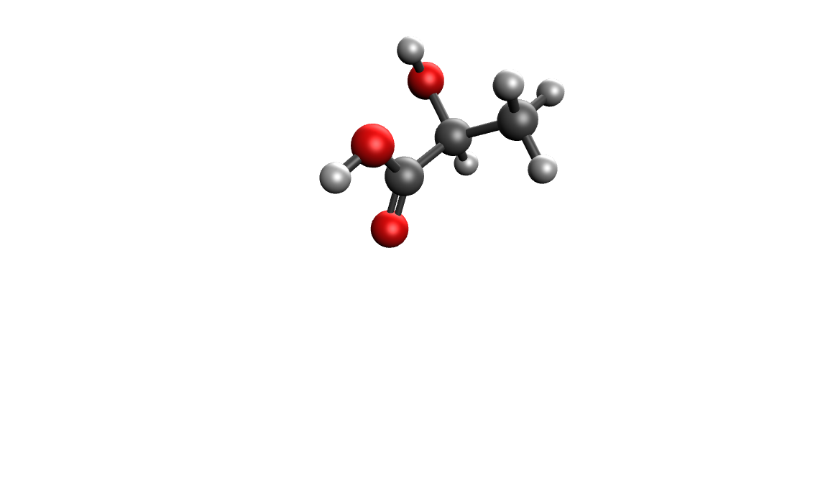


⃝ True ⃝ False

**Written comments**

(+) Describe what you like most about/during the session

(-) What can be improved by the instructor team

(Δ) What you have learned during the session
