# Supplementary material for: A virtual alternative to molecular model sets: a beginners’ guide to constructing and visualizing molecules in open-source molecular graphics software
Source: BMC Res Notes. 2021 Feb 17;14:66. doi: 10.1186/s13104-021-05461-7 (PMC7887714; doi:10.1186/s13104-021-05461-7)
Supplement: Supplementary file 3 — Additional file 3. Survey questions and detailed results. [file 13104_2021_5461_MOESM3_ESM.zip › Survey/pre-and post tests.pdf]

SEC_____ No_____ Date ___________

These are pre-activity questions.
Please do not remove the staple until you are told to do so.

1. What computer did you use for the activity today?
   ◯ Your own computer ◯ The university's computer laboratory

2. What operating system did you use for the activity today?
   ◯ Windows ◯ Mac ◯ Linux

3. A molecule with two lone pairs and two atoms bonded to the central atom has a bent structure.
   ◯ True ◯ False

4. The colours of atomic orbital in MOs correspond to their positive and negative charges.
   ◯ True ◯ False

5. Water is a polar molecule.
   ◯ True ◯ False

6. The molecular structure on the left is represented by the picture on the right.

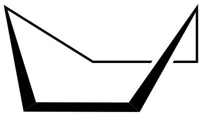

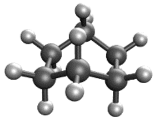

   ◯ True ◯ False

These are post-activity questions.

The software

Avogadro	IQmol
7a. Can you download the program?<br>◯ Yes ◯ No, please specify reason(s)	7b. Can you download the program?<br>◯ Yes ◯ No, please specify reason(s)
8a. Can you install the program?<br>◯ Yes ◯ No, please specify reason(s)	8b. Can you install the program?<br>◯ Yes ◯ No, please specify reason(s)
9a. Can you use the program to complete assigned tasks?<br>◯ Yes ◯ No, please specify reason(s)	9b. Can you use the program to complete assigned tasks?<br>◯ Yes ◯ No, please specify reason(s)

Knowledge

10. A molecule with zero lone pair and 6 atoms bonded to the central atom has an octahedral structure.
◯ True ◯ False

11. The colours of atomic orbital in MOs correspond to the phases of the wavefunction.
◯ True ◯ False

12. Methane is a polar molecule.
◯ True ◯ False

13. The molecular structure on the left is represented by the picture on the right.

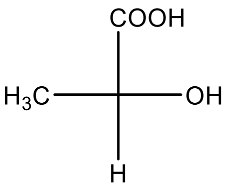

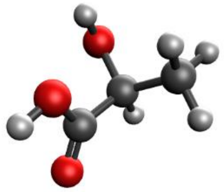

◯ True ◯ False

Written comments

(+) Describe what you like most about/during the session

(-) What can be improved by the instructor team

(Δ) What you have learned during the session

DOI: 10.1186/s13104-021-05461-7
