# Supplementary material for: A virtual alternative to molecular model sets: a beginners’ guide to constructing and visualizing molecules in open-source molecular graphics software
Source: BMC Res Notes. 2021 Feb 17;14:66. doi: 10.1186/s13104-021-05461-7 (PMC7887714; doi:10.1186/s13104-021-05461-7)
Supplement: Supplementary file 3 — Additional file 3. Survey questions and detailed results. [file 13104_2021_5461_MOESM3_ESM.zip › Survey/review questions.pdf]

Circle picture(s) that represent hydrogen atom's *p* orbital. (0.5 point)

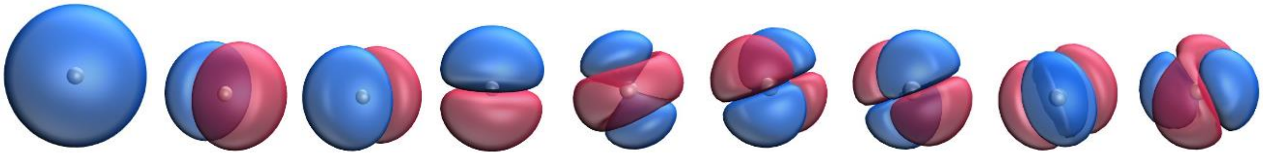

HOMO stands for ____ (0.5 point)

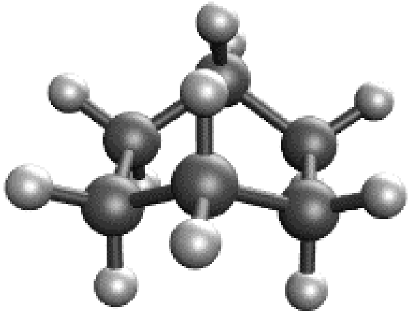

Circle a bond-line structure below that best represent the molecule in the picture. (1 point)
If you cannot find one, circle no solution.

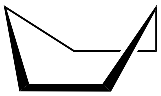

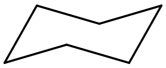

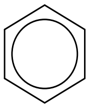

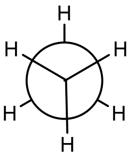

no solution

DOI: 10.1186/s13104-021-05461-7

Circle picture(s) that represent hydrogen atom's *s* orbital. (0.5 point)

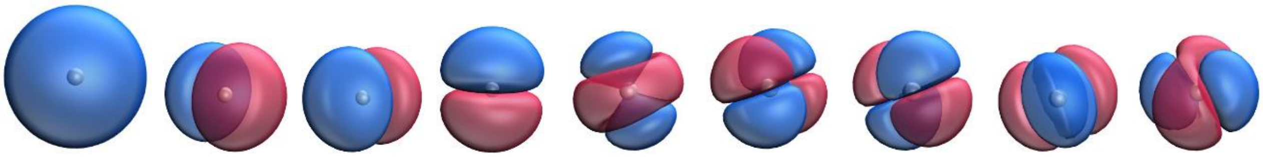

LUMO stands for ____ (0.5 point)

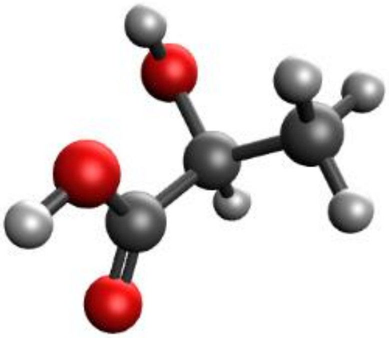

Circle a bond-line structure below that does NOT represent the molecule in the picture. (1 point)
If you cannot find one, circle no solution.

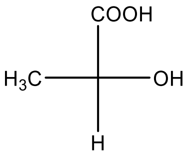

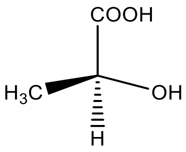

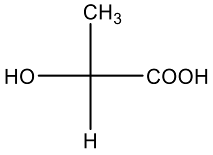

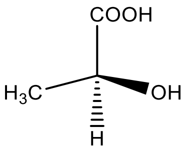

no solution
