# Supplementary material for: A virtual alternative to molecular model sets: a beginners’ guide to constructing and visualizing molecules in open-source molecular graphics software
Source: BMC Res Notes. 2021 Feb 17;14:66. doi: 10.1186/s13104-021-05461-7 (PMC7887714; doi:10.1186/s13104-021-05461-7)
Supplement: Supplementary file 3 — Additional file 3. Survey questions and detailed results. [file 13104_2021_5461_MOESM3_ESM.zip › Survey/Survey_Result_Jan2021.pdf]

		Pre-test						Post-test									
	Student	1	2	3	4	5	6	7a	7b	8a	8b	9a	9b	10	11	12	13
	1	P	M	F	T	T	F	Y	Y	Y	Y	Y	Y	T	F	F	F
	2	P	M	F	T	T	F	Y	Y	Y	Y	Y	Y	T	T	F	F
	3	P	W	T	T	F	T	-	Y	-	Y	-	Y	T	T	F	F
	4	P	M	F	F	T	T	Y	Y	Y	Y	Y	Y	T	F	F	T
	5	P	W	T	T	T	F	-	N	-	N	-	N	T	T	F	F
	6	P	W	T	F	T	F	-	Y	-	Y	-	Y	T	T	F	F
	7	P	M	T	T	T	T	-	I did	-	I ha	-	Tod	T	F	F	F
	8	P	W	T	T	T	T	-	Y	-	Y	-	Y	T	T	F	F
	9	P	M	F	F	T	F	-	Y	-	Y	-	Y	T	T	F	T
	10	P	M	T	F	T	F	-	Y	-	Y	-	Y	T	T	F	T
	11	P	W	T	T	T	T	-	Y	-	Y	-	Y	T	F	F	F
	12	P	W	T	T	T	T	Y	Y	Y	Y	Y	Y	F	F	F	F
	13	P	W	T	T	T	F	-	Y	-	Y	-	Y	T	F	F	T
	14	P	M	T	T	T	F	Y	Y	Y	Y	Y	Y	T	T	F	T
	15	P	M	F	T	T	T	-	N	-	N	-	Y	T	T	F	F
	16	P	M	F	F	T	F	-	Y	-	Y	-	Y	F	F	F	F
	17	P	M	F	T	T	F	Y	Y	Y	Y	Y	Y	T	T	F	F
	18	P	W	F	T	T	T	-	Y	-	Y	-	Y	F	T	F	F
	19	P	W	T	T	T	T	-	Y	Y	Y	N, I	Y	T	T	F	T
	20	P	W	F	F	T	F	-	Y	-	Y	-	Y	T	T	F	F
	21	P	W	T	T	T	F	-	Y	-	Y	-	Y	T	T	F	F
	22	P	M	T	T	T	F	-	Y	-	Y	-	Y	T	T	F	F
	23	P	W	T	F	T	T	-	Y	-	Y	-	Y	F	F	F	F
Total valid		23	23	23	23	23	23	23	23	23	23	23	23	23	23	23	23
P	Personal computer	100%	0%	0%	0%	0%	0%	0%	0%	0%	0%	0%	0%	0%	0%	0%	0%
U	University's computer	0%	0%	0%	0%	0%	0%	0%	0%	0%	0%	0%	0%	0%	0%	0%	0%
B	Personal and univeristy's computer	0%	0%	0%	0%	0%	0%	0%	0%	0%	0%	0%	0%	0%	0%	0%	0%
W	Windows	0%	52%	0%	0%	0%	0%	0%	0%	0%	0%	0%	0%	0%	0%	0%	0%
M	Mac	0%	48%	0%	0%	0%	0%	0%	0%	0%	0%	0%	0%	0%	0%	0%	0%
Y	Yes	0%	0%	0%	0%	0%	0%	26%	87%	30%	87%	26%	91%	0%	0%	0%	0%
N	No	0%	0%	0%	0%	0%	0%	0%	9%	0%	9%	0%	4%	0%	0%	0%	0%
T	TRUE	0%	0%	61%	70%	96%	43%	0%	0%	0%	0%	0%	0%	83%	65%	0%	26%
F	FALSE	0%	0%	39%	30%	4%	57%	0%	0%	0%	0%	0%	0%	17%	35%	100%	74%

DOI: 10.1186/s13104-021-05461-7

For t-test

Student	Pre-test				Sum	Post-test				Sum
1	0	0	1	0	0.25	1	0	1	0	0.50
2	0	0	1	0	0.25	1	1	1	0	0.75
3	1	0	0	1	0.50	1	1	1	0	0.75
4	0	1	1	1	0.75	1	0	1	1	0.75
5	1	0	1	0	0.50	1	1	1	0	0.75
6	1	1	1	0	0.75	1	1	1	0	0.75
7	1	0	1	1	0.75	1	0	1	0	0.50
8	1	0	1	1	0.75	1	1	1	0	0.75
9	0	1	1	0	0.50	1	1	1	1	1.00
10	1	1	1	0	0.75	1	1	1	1	1.00
11	1	0	1	1	0.75	1	0	1	0	0.50
12	1	0	1	1	0.75	0	0	1	0	0.25
13	1	0	1	0	0.50	1	0	1	1	0.75
14	1	0	1	0	0.50	1	1	1	1	1.00
15	0	0	1	1	0.50	1	1	1	0	0.75
16	0	1	1	0	0.50	0	0	1	0	0.25
17	0	0	1	0	0.25	1	1	1	0	0.75
18	0	0	1	1	0.50	0	1	1	0	0.50
19	1	0	1	1	0.75	1	1	1	1	1.00
20	0	1	1	0	0.50	1	1	1	0	0.75
21	1	0	1	0	0.50	1	1	1	0	0.75
22	1	0	1	0	0.50	1	1	1	0	0.75
23	1	1	1	1	1.00	0	0	1	0	0.25
					58%					68%

DOI: 10.1186/s13104-021-05461-7

Student	+	-	Δ
1	Stress	nothing	a lot of chemistry program and structures
2	breakout room because I have to c chance to meet new friends.	explaining the instruction clearly and slowly	a lot of knowledge about chemistry ex. polar bond/ polar molecules and etc
3	During the session, what I like the most could be the part that I used the program called IQmol. I think the program is quite interesting but as a first time using this program, it is quite hard for me to use such as visualizing orbitals.	For today's class, I think it's fine.	I learned how to use the program to draw a basic molecular geometries, visualize the orbitals, find polarity of molecule, and match 3D structure with bond line structure. I learned that IQmol which is one of the program can be used for identifying what I have mentioned before.
4	Using the programs was new and it can be quite fun if we get used to the functions	Maybe demonstrate the examples slower	how to program the molecules using IQmol and Avogadro in order to visualize 3D structures
5	I like when teacher arranged groups for assignment, and take care of students.	Problems about opening files and software during the sessions	I learned how to create chemical structures by using IQmol application, and it helps me to do some review the lessions from Chemistry 1 and 2.
6	Learning how to use program to construct molecular models and exploring what it can do	Maybe provide more examples and slow down a little bit. I was able to catch up before the breakout room but my partner was not quite, so we spent some time going over it again. Anyhow, I think the instructor have done really well and I like how he visited the breakout rooms very often to help and explain in every part we were	I learned how to use IQmol program, construct molecular models , apply the lessons studied in General Chemistry I into the lab, work with someone totally new
7	I probably like group work the most because it's the part where we start sharing ideas, suggesting and discuss with friends.	Everything is great. The instructor team was very helpful. However, it would be great if installing or downloading program into the computer was informed a few days before class.	I learned how to build molecule using IQ mol and get to revise hybridization, orbitals, shape of molecules and more.
8	Collaboration	none	Using the programe to model molecules
9	the instructor is kind and humorous, activity is fun	explain about the assignments and teach students some more basic in order to complete the assigned work	how to use IQ mol, polar and non polar
10	I really like the breakout room in this session. Since there are only two people in the room, it is less awkward.	I think the instructor, sometimes speak very fast and hard to follow.	With the help of IQmol, I now have a very clear picture of the structure the molecules and the orbitals.
11	New program helping learning	Nothing to be improved. It's OK.	molecular structures, molecular forces, orbitals.
12	Nothing.	Please allow us to turn off our cam.	I learnt that I am horrible at Chemistry.
13	instructor is nice	speak more clearly	know how to use IQmol
14	I really like the new application that we have to use for the assignment, this might help me in the future when i want to know the polar, or the shape of compounds.	Should explain more about the tasks, some of them are not quite clear to understand.	Learn about the orbital and polar and how to use the iqmole application.
15	very good take care of student and always help me to complete assignment.	about the technical problem such as avogadro which I cannot download it.	Using IQmol application and corporate with peer in order to get the answer.
16	I like every things in class. this is how I imagine the online class should be and it nice. the work is not that hard core and it's not that easy too which is great.	I think ajarn mic is too quiet. I have to lean in to understand.	molecular structure.
17	trying to figure the chemical molecule in iQmol program	should be an announcement to download the program before class	Working with someone I don't know before and know the shape of orbital in 3 dimention
18	Working with my friends	Maybe more time to do the lab reports	How to use programs to create molecule structures.
19	the demonstration of the molecule	Maybe when instructor introduce new things just do it a little bit slower.	Team work and structure/shape of the molecule
20	Using the IQmol application	I would really appreciate if you can speak a bit slower and louder	Using IQmol
21	Using the program to do tasks.	Slow down, give more time to do work.	Reviewed knowledge from GenChem I, II.
22	Group activity. It was fun to work in pair	The instructor team can go a bit slower. I could not follow some of the parts.	I've learned how to use a new program(iqmol)
23	The programme gives a clear illustration of the molecule's structure	The pace of speech is a little bit fast. It was quite difficult for me to catch up.	How to use the program, how to identify polar or non polar by looking at the structure and the clear images of each molecular orbitals.

DOI: 10.1186/s13104-021-05461-7
