# Supplementary material for: A virtual alternative to molecular model sets: a beginners’ guide to constructing and visualizing molecules in open-source molecular graphics software
Source: BMC Res Notes. 2021 Feb 17;14:66. doi: 10.1186/s13104-021-05461-7 (PMC7887714; doi:10.1186/s13104-021-05461-7)
Supplement: Supplementary file 3 — Additional file 3. Survey questions and detailed results. [file 13104_2021_5461_MOESM3_ESM.zip › Survey/Survey_Result_Sep2020.pdf]

		Pre-test						Post-test									
	Student\Questions	1	2	3	4	5	6	7a	7b	8a	8b	9a	9b	10	11	12	13
	1	U	W	T	F	T	F	Y	Y	Y	Y	Y	Y	T	T	T	T
	2	U	W	T	T	T	F	Y	Y	Y	Y	Y	Y	T	T	T	F
	3	U	W	T	F	T	T	Y	Y	Y	Y	Y	Y	T	F	F	T
	4	U	W	F	T	T	T	Y	Y	Y	Y	Y	Y	T	T	F	F
	5	U	W	T	T	T	T	Y	Y	Y	Y	Y	Y	T	T	T	T
	6	U	W	F	T	F	T	Y	Y	Y	Y	Y	Y	T	T	F	F
	7	U	W	T	T	T	F	Y	Y	Y	Y	Y	Y	T	T	F	F
	8	U	W	F	T	T	F	Y	Y	Y	Y	Y	Y	T	T	F	F
	9	U	W	F	T	T	T	Y	Y	Y	Y	Y	Y	T	F	F	T
	10	U	W	T	F	T	F	Y	Y	Y	Y	Y	Y	T	T	F	T
	11		W	T	F	T	T	Y	Y	Y	Y	Y	Y	T	T	F	F
	12	B	W	F	F	T	T	Y	Y	Y	Y	Y	Y	F	T	F	T
	13	U	W	F	F	T	T	N	Y	N	Y	N	Y	T	F	T	F
	14	U	W	F	T	F	T	Y	Y	Y	Y	Y	Y	T	T	T	F
	15	U	W	T	F	T	F	Y	Y	Y	Y	Y	Y	T	T	F	T
	16	U	W	T	T	T	F	Y	Y	Y	Y	Y	Y	F	T	F	T
	17	U	W	F	F	T	T		Y		Y		Y	T	T	F	F
	18	U	W	F	T	T	F	Y	Y	Y	Y	Y	Y	T	T	F	T
	19	P	W	T	T	T	F	N	Y	N	Y	N	Y	T	T	F	T
	20	P	W	T	F	T	T	N	Y	N	Y	N	Y	T	T	F	F
	21	U	W	T	F	T	F	N	Y	N	Y	N	Y	T	T	T	F
	22	U	W	T	F	T	F		Y		Y		Y	T	T	T	F
	23	U	W	T	F	T	F	Y	Y	Y	Y	Y	Y	T	T	F	F
	24	U	W	T	F	T	T	N	Y	N	Y	N	Y	T	T	F	T
	25	U	W	T	T	T	F	Y	Y	Y	Y	Y	Y	T	T	T	F
	26	U	W	T	T	T	F	Y	Y	Y	Y	Y	Y	T	T	F	T
	27	U	W	F	F	T	T	N	Y	N	Y	N	Y	T	T	F	T
	28	U	W	T	F	T	F	N	Y	N	Y	N	Y		F	F	T
	29	U	W	T	F	T	F	Y	Y	Y	Y	Y	Y	T	T	T	F
	30	U	W	F	T	T	F	Y	Y		Y		Y	T	T	F	T
	31	U	W	T	T	T	F	N	Y	N	Y	N	Y	F	T	F	T
Total valid		30	31	31	31	31	31	29	31	28	31	28	31	30	31	31	31
P	Personal computer	7%	0%	0%	0%	0%	0%	0%	0%	0%	0%	0%	0%	0%	0%	0%	0%
U	University's computer	90%	0%	0%	0%	0%	0%	0%	0%	0%	0%	0%	0%	0%	0%	0%	0%
B	Personal and univeristy's computer	3%	0%	0%	0%	0%	0%	0%	0%	0%	0%	0%	0%	0%	0%	0%	0%
W	Windows	0%	100%	0%	0%	0%	0%	0%	0%	0%	0%	0%	0%	0%	0%	0%	0%
M	Mac	0%	0%	0%	0%	0%	0%	0%	0%	0%	0%	0%	0%	0%	0%	0%	0%
Y	Yes	0%	0%	0%	0%	0%	0%	72%	100%	71%	100%	71%	100%	0%	0%	0%	0%
N	No	0%	0%	0%	0%	0%	0%	28%	0%	29%	0%	29%	0%	0%	0%	0%	0%
T	TRUE	0%	0%	65%	48%	94%	42%	0%	0%	0%	0%	0%	0%	90%	87%	29%	52%
F	FALSE	0%	0%	35%	52%	6%	58%	0%	0%	0%	0%	0%	0%	10%	13%	71%	48%

DOI: 10.1186/s13104-021-05461-7

For t-test

Student	Pre-test				Sum	Post-test				Sum
1	1	1	1	0	0.75	1	1	0	1	0.75
2	1	0	1	0	0.50	1	1	0	0	0.50
3	1	1	1	1	1.00	1	0	1	1	0.75
4	0	0	1	1	0.50	1	1	1	0	0.75
5	1	0	1	1	0.75	1	1	0	1	0.75
6	0	0	0	1	0.25	1	1	1	0	0.75
7	1	0	1	0	0.50	1	1	1	0	0.75
8	0	0	1	0	0.25	1	1	1	0	0.75
9	0	0	1	1	0.50	1	0	1	1	0.75
10	1	1	1	0	0.75	1	1	1	1	1.00
11	1	1	1	1	1.00	1	1	1	0	0.75
12	0	1	1	1	0.75	0	1	1	1	0.75
13	0	1	1	1	0.75	1	0	0	0	0.25
14	0	0	0	1	0.25	1	1	0	0	0.50
15	1	1	1	0	0.75	1	1	1	1	1.00
16	1	0	1	0	0.50	0	1	1	1	0.75
17	0	1	1	1	0.75	1	1	1	0	0.75
18	0	0	1	0	0.25	1	1	1	1	1.00
19	1	0	1	0	0.50	1	1	1	1	1.00
20	1	1	1	1	1.00	1	1	1	0	0.75
21	1	1	1	0	0.75	1	1	0	0	0.50
22	1	1	1	0	0.75	1	1	0	0	0.50
23	1	1	1	0	0.75	1	1	1	0	0.75
24	1	1	1	1	1.00	1	1	1	1	1.00
25	1	0	1	0	0.50	1	1	0	0	0.50
26	1	0	1	0	0.50	1	1	1	1	1.00
27	0	1	1	1	0.75	1	1	1	1	1.00
28	1	1	1	0	0.75	0	0	1	1	0.50
29	1	1	1	0	0.75	1	1	0	0	0.50
30	0	0	1	0	0.25	1	1	1	1	1.00
31	1	0	1	0	0.50	0	1	1	1	0.75
					63%					74%

DOI: 10.1186/s13104-021-05461-7

Student	+	-	Δ
1	Working on computer	Information on the programs can be slowed down.	How to use programs for work.
2	Iqmol is easy to use	I don't want to improve.	How to use IQmol.
3	A-Z part + Excel	See more if students are following (how to use Avogadro + IQmol instruction is too fast) + computer = window, instructor = mac - different version.	Excel, Avogadro, IQmol, shape, color…
4	I like the way that we use program. It make me get more images of molecular geometry of molecules.	It does not have anything to improve much, but just only the way that they describe the method should be slower.	I have learned how to put graphs in excel which I have never done before. Also, I have a chance to use IQmol which is used for construct the molecular geometry of molecules.
5	See the structure of the molecule on program.	Teach steps a bit slower.	About structure, polarity, MO.
6		Less assignment.	Using IQmol.
7	Using computer.	Time management.	Using iqmol and avogadro.
8	We can see the structure of whatever we want to see.	Sometime it's too fast to explain.	How to work on with the software.
9	Have many person to ask a question.		How to use Avogadro and IQmol.
10	I like the program very much, it help me visualize molecular structure better.	I think it is better for student to fill in the worksheet after class.	How to visualize molecular structure and understand its function better.
11	Doing work with partner and learning how to use Iqmol and Avogadro.	I felt like the instructor was speaking too fast. I can't catch up sometimes.	I learn how to use program and recap my knowledge from gen chem I.
12	Good program.	More organized.	How to build small balls.
13	Discussing the molecular structure with partner, so we could share our ideas and knowledge.	Lessen task due to the time limit and for some students who are not good at technology, the time limitation should be longer or the work should be lesser.	Learn about the bonding structure of molecules.
14	The thing that I like the most is that I am using the programme to see the chemical structure.	The teaching might be slower and have a blittle more time gap for students to work.	I have learned about to see the structure in 3D.
15	Building molecules.		I learned more about shapes of
16	IQmol program, work with pair, consumed-energy activity.	slower & more detail instructors.	learn how to use IQmol.
17	There are lab assistant that help describing thing for us which make me understand more about assignment.	Instructor may speak while describing about program and assignments.	I have learned about how to use IQmol program and drawing molecule structure in a program.
18	When doing work with the partner.	Too much work load for today.	Mostly the structure part (How to analyze).
19	3D model of molecule.	Pre download the software.	I learned how to do the model of molecule in 3D program.
20	I like that we use computer to model chemical structures.	There are a lots of things to do and get familiar with so it confuses me a bit.	I learned how to use Iqmol.
21	We are able to use the program to see the structure of the molecules and can visualize the molecules better.	Go through the process more slowly.	How to use IQmol and revision of VSEPR model.
22	I really like the program.	Nothing	The structure of molecule.
23	I liked making the models using the program IQmol.	As I was working on task 1, the professor was already explaining task 2, so it was a little fast. If the lecture was paced slower, it might have been more efficient.	How ot use Iqmol and how to show polarity, orbitals etc.
24	Using IQmol to see the molecule structure.	Unclear solution to a question: 4.Haworth structure.	How to use IQmol software to visualize molecular structures and to help determine the molecules' polarity.
25	Try to make a model or may be the task 3.Polarity part, I like to see the arrow.	Nothing	Learn to make models.
26	The new software I was introduced to use.	May be to lower the amount of tasks.	I learnt about how to use the new software to identify molecules using VSEPR.
27	There is senior to help us out when we couldn't follow the teacher.		The model of molecular structure of many types of molecules.
28	Relax environment, comfortable asking questions.	list of tasks in a written form.	How to use iqmol, visually see the shape of each molecule.
29	The program is interesting.	None	To use the IQmol program.
30			I learn how to use Iqmol program and the shape of molecules.
31	I like the experience of seeing the 3D shape structure of the molecule through computer program.	I would appreciate if the instruction could be explain slower.	I have learned how to use IQmol program.

DOI: 10.1186/s13104-021-05461-7
